# Remote Monitoring and Behavioral Economics in Managing Heart Failure in Patients Discharged From the Hospital: A Randomized Clinical Trial

**DOI:** 10.1001/jamainternmed.2022.1383

**Published:** 2022-05-09

**Authors:** David A. Asch, Andrea B. Troxel, Lee R. Goldberg, Monique S. Tanna, Shivan J. Mehta, Laurie A. Norton, Jingsan Zhu, Lauren G. Iannotte, Tamar Klaiman, Yuqing Lin, Louise B. Russell, Kevin G. Volpp

**Affiliations:** 1 Department of Medicine, Perelman School of Medicine, University of Pennsylvania, Philadelphia; 2 Leonard Davis Institute of Health Economics, University of Pennsylvania, Philadelphia; 3 Department of Medical Ethics and Health Policy, Perelman School of Medicine, University of Pennsylvania, Philadelphia; 4 Division of Biostatistics, NYU Grossman School of Medicine, New York, New York

## Abstract

**Importance:**

Close remote monitoring of patients following discharge for heart failure (HF) may reduce readmissions or death.

**Objective:**

To determine whether remote monitoring of diuretic adherence and weight changes with financial incentives reduces hospital readmissions or death following discharge with HF.

**Design, Setting, and Participants:**

The Electronic Monitoring of Patients Offers Ways to Enhance Recovery (EMPOWER) study, a 3-hospital pragmatic trial, randomized 552 adults recently discharged with HF to usual care (n = 280) or a compound intervention (n = 272) designed to inform clinicians of diuretic adherence and changes in patient weight. Patients were recruited from May 25, 2016, to April 8, 2019, and followed up for 12 months. Investigators were blinded to assignment but patients were not. Analysis was by intent to treat.

**Interventions:**

Participants randomized to the intervention arm received digital scales, electronic pill bottles for diuretic medication, and regret lottery incentives conditional on the previous day’s adherence to both medication and weight measurement, with $1.40 expected daily value. Participants’ physicians were alerted if participants’ weights increased 1.4 kg in 24 hours or 2.3 kg in 72 hours or if diuretic medications were missed for 5 days. Alerts and weights were integrated into the electronic health record. Participants randomized to the control arm received usual care and no further study contact.

**Main Outcomes and Measures:**

Time to death or readmission for any cause within 12 months.

**Results:**

Of the 552 participants, 290 were men (52.5%); 291 patients (52.7%) were Black, 231 were White (41.8%), and 16 were Hispanic (2.9%); mean (SD) age was 64.5 (11.8) years. The mean (SD) ejection fraction was 43% (18.1%). Each month, approximately 75% of participants were 80% adherent to both medication and weight measurement. There were 423 readmissions and 26 deaths in the control group and 377 readmissions and 23 deaths in the intervention group. There was no significant difference between the 2 groups for the combined outcome of all-cause inpatient readmission or death (unadjusted hazard ratio, 0.91; 95% CI, 0.74-1.13; *P* = .40) and no significant differences in all-cause inpatient readmission or observation stay or death, all-cause cardiovascular readmission or death, time to first event, and total all-cause deaths. Participants in the intervention group were slightly more likely to spend fewer days in the hospital.

**Conclusions and Relevance:**

In this randomized clinical trial, there was no reduction in the combined outcome of readmission or mortality in a year-long intensive remote monitoring program with incentives for patients previously hospitalized for HF.

**Trial Registration:**

ClinicalTrials.gov Identifier: NCT02708654

## Key Points

**Question:**

Among patients discharged after a hospitalization for heart failure, does remote monitoring and rewarding their weight and diuretic adherence reduce subsequent chances of death or rehospitalization?

**Findings:**

In this randomized clinical trial of 552 adults followed up for 12 months, hospital readmissions or death were not significantly different whether patients received remote monitoring and financial incentives or usual care.

**Meaning:**

In this randomized clinical trial, a comprehensive remote monitoring system with incentives did not improve patient outcomes in patients with heart failure discharged from the hospital.

## Teaser

This randomized clinical trial examines the use of remote monitoring of weight and adherence to diuretic therapy in patients with heart failure after hospital discharge.

## Introduction

Heart failure (HF) management depends on a complex array of medications, lifestyle changes, follow-up care, and patient participation: a few missed doses of a diuretic medication or a small increase in dietary sodium can result in readmission.^1,2^ Clinicians need timely awareness of often subtle changes in weight or adherence to intervene before clinical deterioration, and yet those indicators are typically out of view.^3,4^ The result is a combination of awareness of the many potential opportunities for intervention along the pathway to HF readmission, and yet no efficient approaches to implement them.^5,6,7,8,9,10^

A hope has been to engage patients in self-monitoring and efficiently engage clinicians in the care of those patients outside of office visits. New insights from the field of behavioral economics offer promise for sustaining this kind of patient engagement, for example, by offering rewards that not only provide immediate incentives for adherence but that are designed to harness known behavioral tendencies.^11^ Daily lotteries have improved medication adherence and weight loss, and those that incorporate anticipated regret (people are notified whether they won or would have won) and variable rewards (frequent small payoffs and infrequent large payoffs) have been demonstrated to be engaging in a variety of contexts.^12^ In addition, new technology allows important information from remote monitoring to be automatically integrated into the electronic health record (EHR), simplifying clinical workflows and presenting data in the context of care.

Herein we report the main results of the Electronic Monitoring of Patients Offers Ways to Enhance Recovery (EMPOWER) trial, a pragmatic randomized clinical trial to evaluate whether an automated approach to patient engagement that incorporates behavioral economic principles can reduce readmissions in patients with HF discharged from the hospital.

## Methods

### Overview

The EMPOWER trial was a 2-arm pragmatic randomized clinical trial comparing usual care in the management of patients discharged from the hospital after an admission for HF with a compound intervention comprising (1) daily automated assessments of weight and adherence to oral diuretic medications, (2) financial incentives and a support partner to encourage those assessments, and (3) reporting of substantial weight changes or nonadherence to diuretics to the managing clinician in the EHR. Participants provided verbal informed consent and received financial compensation. Patients were enrolled after discharge from a hospitalization for HF and were followed up for 1 year. The protocol and statistical analysis plan are reported in Supplement 1 and elsewhere.^13^ The study was approved by the institutional review board of the University of Pennsylvania. This study followed the Consolidated Standards of Reporting Trials (CONSORT) reporting guideline.

### Participants, Recruitment, and Randomization

Participants were recruited between May 25, 2016, and April 8, 2019. Eligible participants were aged 18 to 80 years, discharged from 1 of 3 Penn Medicine hospitals in Philadelphia, Pennsylvania, with either a primary diagnosis of HF (preserved or reduced ejection fraction), a secondary diagnosis of HF with intravenous diuretics administered during their inpatient stay, or HF appearing on their problem list with intravenous diuretics administered during their hospital stay. Participants were eligible to enroll up to 30 days postdischarge if prescribed a daily diuretic and expected to have HF followed up by a Penn Medicine clinician (cardiology or primary care). Participants were excluded if they had kidney failure, required inotrope therapy, were listed for or receiving a heart transplant or ventricular assist device, were receiving palliative or hospice care, had a history of uncontrolled cognitive or psychiatric conditions that could affect study participation, or were participating in another telemonitoring program.

We enrolled patients with both preserved and reduced ejection fractions. Clinical practice guidelines recommend that all patients with HF be enrolled in a disease management program.^14^ For both diseases, similar lifestyle changes and monitoring are recommended.

Potentially eligible participants received up to 5 telephone calls from study staff. Participants providing verbal informed consent were then randomized to intervention or control in a 1:1 ratio stratified by hospital site. Investigators and data analysts were blinded to arm assignment; patients, study staff, and treating clinicians were not blinded. All enrolled participants received $25 for participation.

### Study Arms

Participants randomized to the usual care arm received no further engagement with study personnel. Participants randomized to the intervention arm received (1) a digital scale; (2) an electronic pill bottle (LLC Technologies) to use for diuretic medication; (3) daily regret lottery incentives with an 18% chance of a $5 payout and a 1% chance of a $50 payout (expected daily value, $1.40) conditional on adherence to both medication and weight measurement from the previous day. Regret lotteries are seen as more motivating than regular lotteries because participants are informed of the prize they would have won had they been adherent the day before and anticipate and seek to avoid the regret of missing a prize.^15,16^ Participants were also invited to identify a support partner who, with agreement, would receive alerts about nonadherence to remote monitoring. Support partners might directly encourage participant adherence or enhance it by creating known witnesses to it.^17,18^

In the intervention arm, participants’ weights were automatically assessed against prespecified weight change thresholds of either 1.4 kg in 24 hours or 2.3 kg in 72 hours. Patients crossing either threshold were contacted by study staff to verify weights and answer a symptom questionnaire. Verified information was then posted as an abnormal result into the EHR and routed to the patient’s managing clinician. Weekly, all verified weights were automatically imported into an EHR flowsheet in the participant’s record.

Participants not weighing themselves or opening their pill bottle for 2 days received an automated message encouraging adherence; support partners also received the message. After 3 days of nonadherence, study staff called the participant; after 4 days, study staff called the support partner. After 5 days of nonadherence, study staff alerted the participant’s clinicians through the EHR.

### Study Outcomes

The primary outcome was time to death or readmission for any cause. Outcomes were observed using EHR data from Penn Medicine, along with all-payer state data on inpatient admissions from Pennsylvania, New Jersey, and Delaware. Secondary outcomes included cardiovascular-related admissions or death; all-cause readmission, death, or observation stay; adherence to study devices; and alerts and responses to them. We performed sensitivity analyses by studying time to first patient event instead of analyzing repeated events per patient and additional outcomes including total number of days in the hospital.

### Statistical Analysis

The trial was designed to provide approximately 80% power, using a 2-sided significance level of .05, to detect a hazard ratio (HR) of 0.73 for the primary outcome (time to death or readmission for any cause). Preliminary data indicated an expected primary event rate at 1 year of approximately 47% in the control group; thus, the trial had approximately 80% power to detect a reduction in the primary event rate of 10 percentage points, to 37% in the intervention group. The primary hypothesis was tested with an unadjusted Andersen-Gill formulation of the standard Cox proportional hazards regression model to properly incorporate repeated events (ie, hospitalizations) within the same individual and account for their correlation.^19,20^ In sensitivity analysis, we applied standard Cox proportional hazards regression models to estimate HRs for time to first event. We also reported models adjusted for self-reported race and ethnicity, age, body mass index, ejection fraction greater than or equal to 40%, and income, using backward elimination augmented with a standard change-in-estimate criterion, successively removing the least significant variable (other than the trial arm) until all remaining variables are significant or they cause a change in the estimate of arm by more than 10%.^21^

To estimate total hospital days, we applied generalized linear models with a Poisson link function. We also assessed the likelihood of future events (readmission or death in the next 1, 2, or 4 weeks) as a function of earlier alerts using multivariate Cox proportional hazards regression models fitted on data from patients in the intervention group. To assess subgroup effects, we interacted arm and a dichotomized indicator for baseline risk factors of interest (eg, ejection fraction at least 35%). Analyses were performed with SAS, version 9.4 (SAS Institute Inc).

## Results

### Study Population

**Figure 1.**
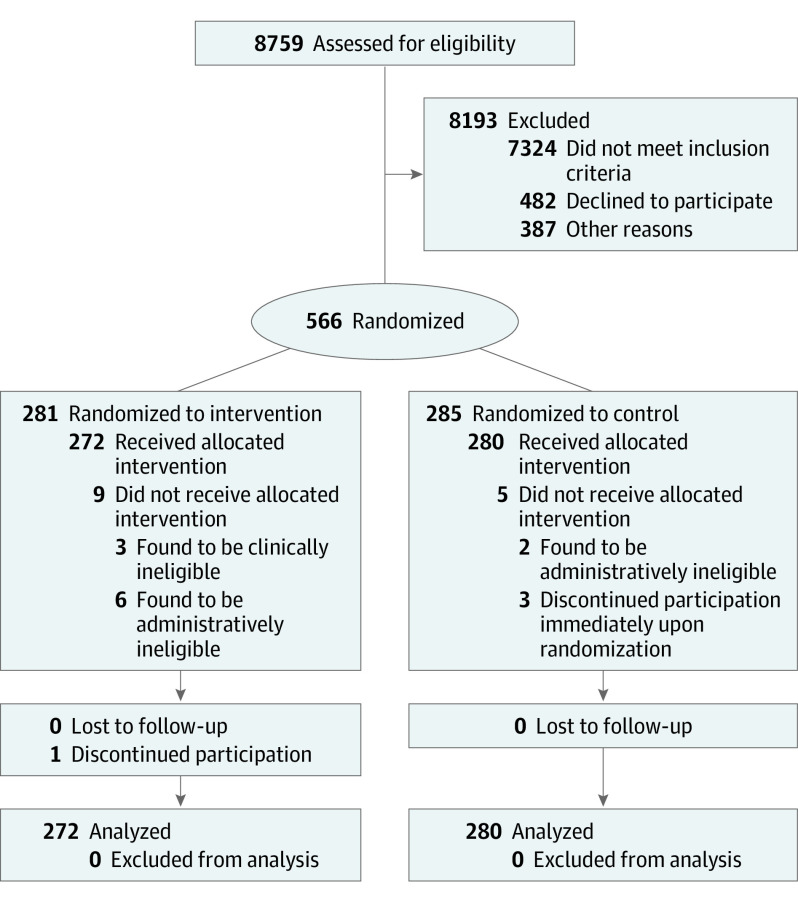
Flow of Participants in the Electronic Monitoring of Patients Offers Ways to Enhance Recovery (EMPOWER) Randomized Clinical Trial

A total of 566 patients were initially deemed eligible and randomly assigned (Figure 1; eTable 1 in Supplement 2). Nine patients assigned to the intervention group and 5 patients assigned to the control group were removed from the study within 24 hours because new information revealed them to be ineligible clinically (eg, kidney function below threshold for inclusion) or administratively (eg, enrolled in a competing trial), or because they withdrew their consent immediately following randomization. Thus, 552 patients were allocated to the intervention (n = 272) or usual care (n = 280) cohorts. One patient assigned to the intervention group discontinued participation later in the study and contributed information up to that point. Patients’ mean (SD) age was 64.5 (11.8) years; 262 were women (47.5%) and 290 were men (52.5%); 291 patients (52.7%) were Black, 231 were White (41.8%), and 30 patients reported other races or declined to provide information; 16 were Hispanic (2.9%). The mean (SD) ejection fraction was 43.0% (18.1%). There were no substantial differences in patient characteristics across the 2 arms (Table 1). Physicians with patients in the study had a mean (SD) of 3.1 (4.4) and a median of 2 patients in the study.

**Table 1.** Participant Characteristics

Characteristic	No. (%)	
	Intervention (n = 272)	Control (n = 280)
Days between index discharge and enrollment, mean (SD)	11.99 (7.37)	12.35 (7.57)
Age, mean (SD), y	64.86 (12.39)	64.23 (11.21)
Sex		
Female	139 (51.1)	123 (43.9)
Male	133 (48.9)	157 (56.1)
Race		
Black	151 (55.5)	140 (50.0)
White	112 (41.2)	119 (42.5)
Other or declined to report	9 (3.3)	21 (7.7)
Hispanic ethnicity	7 (2.8)	9 (3.2)
Self-reported annual income, $		
0-20 000	61 (22.4)	81 (28.9)
>20 000-40 000	38 (14.0)	41 (14.6)
>40 000-60 000	27 (9.9)	23 (8.2)
>60 000-80 000	17 (6.3)	23 (8.2)
≥80 000	26 (9.6)	29 (10.4)
Missing	103 (37.9)	83 (29.6)
Insurance		
Commercial	61 (22.4)	70 (25.0)
Medicaid	46 (16.9)	53 (18.9)
Medicare	164 (60.3)	157 (56.1)
Self-pay	1 (0.4)	0
Body mass index		
<25	49 (18.0)	56 (20.0)
25-34.9	128 (47.1)	114 (40.7)
≥35	95 (34.9)	110 (39.3)
Ejection fraction		
Mean (SD)	42.43 (17.96)	43.95 (18.16)
≥40%	153 (56.3)	166 (59.3)
No. of comorbidities, mean (SD)	4 (1.9)	4 (2.1)
Most prevalent comorbidities^a^		
Hypertension	123 (49.8)	122 (48.8)
Diabetes	112 (45.3)	133 (53.2)
Valvular disease	95 (38.5)	102 (40.8)
Chronic kidney disease	96 (38.9)	89 (35.6)
Obesity	87 (35.2)	97 (38.8)

^a^ Because of missing data for comorbidities, intervention estimates are based on n = 247 and control estimates are based on n = 250.

### Primary End Points

**Figure 2.**
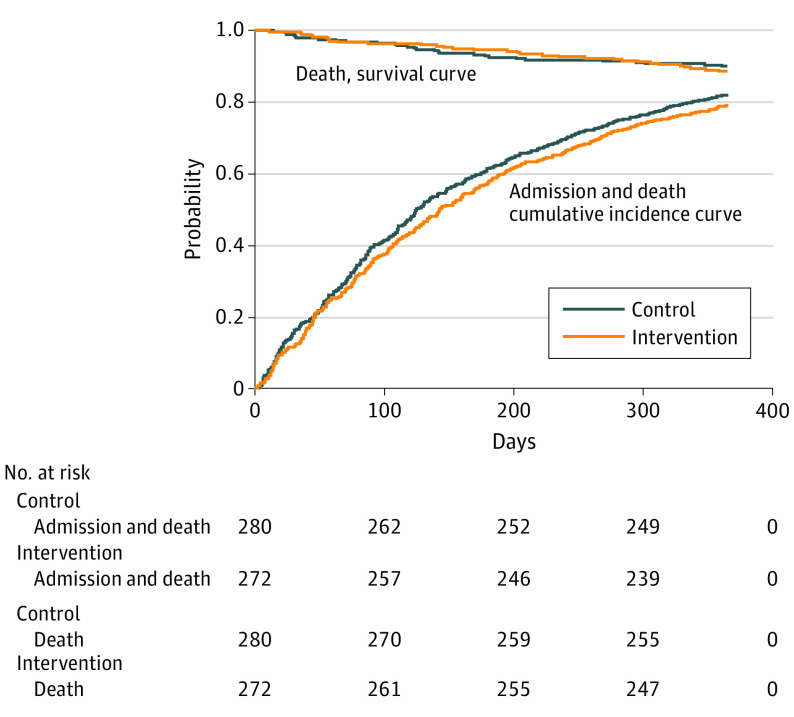
Cumulative Incidence of All-Cause Readmissions or Death

There were 423 (230 related to cardiovascular causes) readmissions and 26 deaths in the control group and 377 (206 related to cardiovascular causes) readmissions and 23 deaths in the intervention groups; 178 patients in the control group (64%) and 171 patients in the intervention group (63%) had at least 1 event. There was no significant difference between the 2 groups for the combined outcome of all-cause inpatient readmission or death (unadjusted HR, 0.91; 95% CI, 0.74-1.13; *P* = .40). The results were essentially the same when observation admissions were included in the outcome, when only admissions for cardiovascular causes were included, and when analyses were adjusted for patient characteristics (Table 2). Figure 2 reveals no separation between the 2 groups in the cumulative incidence of the 2 components (mortality and readmission) of the primary outcome.

**Table 2.** Estimated HRs Comparing Intervention With Control Group for Primary and Secondary Outcomes

HR type	All-cause				Cardiovascular cause readmission or death	
	Inpatient readmission or death		Readmission or observation stay or death			
	HR (95% CI)	*P* value	HR (95% CI)	*P* value	HR (95% CI)	*P* value
Unadjusted	0.91 (0.74-1.13)^a^	.40	0.89 (0.72-1.1)	.29	0.98 (0.77-1.24)	.87
Adjusted	0.90 (0.72-1.12)	.34	0.88 (0.71-1.11)	.26	0.93 (0.73-1.19)	.56

Abbreviation: HR, hazard ratio.

^a^ Primary outcome.

### Secondary End Points

There were no statistically significant differences between the 2 groups in time-to-first-event only (all-cause inpatient readmission or death, all-cause inpatient observation stay or death, and cardiovascular-related inpatient readmission or death), and total all-cause deaths. Patients in the intervention group were slightly more likely to spend fewer days in the hospital (HR, 0.94; 95% CI, 0.9-0.99) (Table 3).

**Table 3.** Estimated HRs Comparing Intervention With Control Group for Additional Outcomes

HR type	First inpatient (all-cause)				First cardiovascular inpatient readmission and death		Total No. days in hospital		Death (all-cause)^a^	
	Readmission, death		Readmission, observation stay, death							
	HR (95% CI)	*P* value	HR (95% CI)	*P* value	HR (95% CI)	*P* value	Days (95% CI)	*P* value	HR (95% CI)	*P* value
Unadjusted	0.95 (0.77-1.17)	.62	1.02 (0.9-1.16)	.73	0.98 (0.77-1.25)	.89	0.94 (0.9-0.99)	.02	1.14 (0.68-1.9)	.62
Adjusted	0.94 (0.75-1.17)	.57	1.00 (0.88-1.15)	.93	0.97 (0.76-1.24)	.81	0.95 (0.9-1)	.09	1.11 (0.66-1.88)	.69

Abbreviation: HR, hazard ratio.

^a^ In all time to first-event studies, death was counted as a censoring event.

There were no significant differences in effects when comparing patients with preserved vs reduced ejection fractions (HR for interaction, 0.72; 95% CI, 0.46-1.11; *P* = .13); results leaned toward greater effect for patients with preserved ejection fraction. Nor were there subgroup differences based on initial body mass index or on recruitment in the first half of the study vs the second. Mean weight change since the start of the study assessed with weights collected similarly in intervention and control groups through routine care was 0 for both the intervention and control groups (eFigure 1 in Supplement 2).

### Intervention Process Measures

Adherence to medication or to weight measurement could be assessed only in the intervention group and ranged from approximately 80% at the start of the observation period to 60% toward the end (eFigure 2 in Supplement 2). Each month, approximately 75% of the participants were 80% adherent to both medication and weight measurement (eTable 2 in Supplement 2). Two hundred sixty intervention participants (95.6%) identified a support partner; 165 partners received alerts about nonadherence to medication or weight measurement (median, 8 alerts; IQR, 3-27).

Of the 261 patients in the intervention group who set up study devices, 237 generated 3736 EHR alerts to clinicians about weight change (median, 10; IQR, 3-21) and 85 alerts about nonadherence (median, 1; IQR, 1-2). Essentially all alerts were opened, and 34.4% of weight change alerts were followed up by a physician response documented in the EHR; 259 alerts indicated chest pain or shortness of breath; 651 were associated with symptoms such as worsening of swelling, decreased appetite, nausea, or difficulty with medications; and 198 indicated other symptoms. The remainder of the participants either had no symptoms or were not reachable. Patients indicating chest pain or shortness of breath had a 1-week cardiovascular event rate of 6.95% compared with 0.50% among those generating alerts with no symptoms reported (eTable 3 in Supplement 2).

Compared with patients generating no alert, the risk-adjusted HRs of all-cause readmission or death for those generating an alert were 2.95 (95% CI, 2.33-3.73; *P* < .001) within 1 week, 3.66 (95% CI, 2.99-4.48; *P* < .001) within 2 weeks, and 4.60 (95% CI, 3.81-5.55; *P* < .001) within 4 weeks. The Harrell C statistic in the risk-adjusted model for first readmission or death within 1 week of the alert was 0.753 if alerts were included in the model, compared with 0.599 if alerts were not included in the model, suggesting that alerts contribute substantial information to the prediction of later clinical events.

## Discussion

We aimed to reduce readmissions or death among patients discharged from the hospital after an admission for HF. We used a comprehensive intervention combining daily remote monitoring of weight and diuretic adherence, state-of-the-art behavioral economic techniques to encourage that monitoring, and low-friction ways to communicate potential patient problems to clinicians in the EHR. The patients in the intervention group did no better than those receiving usual care.

Each of the components of the intervention was executed mostly as planned. Patients set up the remote monitoring devices. They largely maintained engagement, although that engagement decreased over 12 months. Alerts were read by monitoring clinicians, and a third of the alerts were responded to. No step along this pathway was executed perfectly, but this trial was pragmatic—testing a highly complex intervention in the context of usual care.^22^

The results suggest that the combination of elements in this intervention when executed at this level of success was insufficient to alter these patients’ trajectories. Perhaps trial enrollment and device set up occurred too late following the index discharge. Many HF readmissions occur in the early days following discharge; although the timing of trial enrollment was the same in both arms, if earlier admissions would be differentially prevented by the intervention, an overall effect could have been missed. Maybe adherence to diuretics or weight reporting needed to be even higher. Patient adherence was associated with outcome (eTable 2 in Supplement 2), but these associations can be attributed to a healthy user effect in which adherence is not the cause of better outcomes but is instead a marker for health-promoting behaviors more directly on the causal pathway toward them.^23^ Nevertheless, the adherence seen in this study is lower than that previously associated with better HF outcomes.^24^ Maybe the weight change thresholds were too insensitive. Perhaps clinicians responded to too few of the alerts, too late, or incorrectly. Or maybe the intervention was targeted at too broad a patient population—taking in, as it did, a generally unselected heterogeneous set of patients hospitalized with HF, whether with preserved or reduced ejection fraction. More generally, given that this study and another^12^ revealed that many readmissions following discharge for HF are not related to HF (eTable 4 in Supplement 2), it is possible that interventions specifically targeted toward HF are too narrow.

We can speak to some of these issues. The alerts themselves were associated with increases in the events constituting our primary outcome. Although the generation of alerts is a postrandomization variable and such estimates can be subject to omitted variable bias, this finding is consistent with an earlier study suggesting that increasing weight is a risk factor for imminent HF hospitalization.^4^ In addition, we found that adding alerts to the risk-adjusted model increased the model’s discriminating power in predicting short-term events, so use of alerts as a component of a risk prediction algorithm may be warranted. These findings suggest that too few alerts may have prompted clinical responses.

Most individual trials of structured telephone support have similarly not shown a benefit in outcomes; however, a Cochrane meta-analysis reported a significant reduction in HF-related hospitalizations and all-cause mortality.^25^ Most HF centers, including ours, incorporate such structured telephone follow up after hospitalization, and it is possible that our intervention did not show a significant benefit given this and other interventions that are already in place, resulting in a low event rate in both groups. Other studies of noninvasive remote monitoring in HF have shown conflicting results, likely owing to heterogeneity in remote monitoring devices and protocols as well as differences in patient populations, with most individual trials failing to show benefit.^10,26,27^

### Limitations and Strengths

This study has limitations. First, it was conducted in a single academic health system, although across 3 hospitals and among a diverse set of patients. Second, the participants were recruited and observed over a 4-year period when great effort was being introduced into usual care to reduce readmissions and improve guideline-directed medical care for HF. However, we found no differences in effect size for patients recruited earlier in the study compared with later.

This study also has strengths. It was a pragmatic randomized clinical trial against usual care. It captured a relevant patient population at a time that could have plausibly made a difference. It used the study period’s best available technology and clinical and behavioral insights and the execution of the logistic elements succeeded. The reporting of alerts and daily weights was streamlined, leveraging the EHR and simplifying workflows for clinicians.

## Conclusions

In this randomized clinical trial, we found no reduction in the combined outcome of readmission or mortality in this year-long intensive remote monitoring program with incentives for patients previously hospitalized for HF. Success toward this goal may require earlier or deeper patient engagement or expanding engagement to encompass the many non-HF reasons that prompt readmissions of patients with HF.

## Notes

Supplement 1. Trial Protocol and Statistical Analysis Plan

Supplement 2. **eFigure 1.** Patient Weight Change Since Index Hospitalization by Study Group **eFigure 2.** Mean Weekly Adherence to Medication and Weigh-ins for Intervention Group Patients **eTable 1.** Detailed List of Participant Exclusions **eTable 2.** Event Rates According to Degree of Adherence **eTable 3.** Association Between Alerts and Cardiovascular Events at 1, 2, and 4 Weeks for Intervention Group Patients **eTable 4.** Noncardiac Reasons for Admission Among Intervention and Control Group Patients

Supplement 3.
Data Sharing Statement
