# Supplementary material for: Remote Monitoring and Behavioral Economics in Managing Heart Failure in Patients Discharged From the Hospital: A Randomized Clinical Trial
Source: JAMA Intern Med. 2022 May 9;182(6):643–9. doi: 10.1001/jamainternmed.2022.1383 (PMC9171555; doi:10.1001/jamainternmed.2022.1383)
Supplement: Supplement 1. — Trial Protocol and Statistical Analysis Plan [file jamainternmed-e221383-s001.pdf]

Supplement 1

Trial Protocol and Statistical Analysis Plan

David A. Asch, MD, Andrea B. Troxel, ScD, Lee R. Goldberg, MD, Monique S. Tanna, MD, Shivan J. Mehta, MD, MBA, MSHP, Laurie A. Norton, MA, Jingsan Zhu, MS, MBA, Lauren G. Iannotte BA, Tamar Klaiman, PhD, Yuqing Lin, MS, Louise B. Russell, PhD, Kevin G. Volpp, MD, PhD. Remote monitoring and behavioral economics in managing heart failure in patients discharged from the hospital: a randomized clinical trial

	Page
Original protocol	4
Final protocol	20
Summary of protocol changes	44
Original statistical analysis plan	53
Final statistical analysis plan	54
Summary of statistical analysis plan changes	55

Original Study Protocol

Table of Contents

1. Abstract 4

2. Background 4

3. Overall Objectives 5

4. Aims 5

4.1. Primary Aims 5

4.2. Secondary Aims 5

5. Primary Outcome Variable 5

6. Secondary Outcome Variable(s) 5

7. Study Design 5

7.1. Phase 6

7.2. Design 6

7.3. Study Duration 7

7.4. Facilities 7

7.5. Key Inclusion Criteria 8

7.6. Key Exclusion Criteria 8

8. Subject Recruitment 8

8.1. Target Population 8

8.2. Subjects at Penn 8

8.3. Accrual 8

8.4. Patient Subject Recruitment 9

8.5. Subject Compensation 9

9. Study Procedures 9

9.1. Consent Process 9

9.1.1. Waiver of Alerteration of Informed Consent 10

9.1.2. Minimal Risk 10

9.1.3. Impact on Subject Rights and Welfare 10

9.1.4. Waiver Essential to Research 10

9.1.5. Written Statement of Research 10

9.2. Procedures 10

10. Analysis Plan 12

11. Subject Confidentiality 13

11.1. Subject Privacy 13

11.2. Data Disclosure 14

11.3. Data confidentiality 14

12. Consent Process Overview 14

12.1. Potential Study Risks 15

12.2. Potential Study Benefits 15

12.3. Alertnatives to Participation 15

12.4. Data Safety and Monitoring .... **Error! Bookmark not defined.**

Remote monitoring and behavioral economics in managing patients discharged from the hospital with heart failure: a randomized clinical trial

1. Abstract

Using a 2-arm, randomized, controlled trial (RCT) among CHF patients at the University of Pennsylvania Health System (UPHS), we propose to test the effectiveness of applying automated hovering to improve outcomes among CHF patients at high risk of readmission. To conduct this trial we will leverage the Penn Data Store, an electronic repository that can be used to identify study cohorts, and the NIH-funded Way to Health platform, which provides linkages between home-based remote monitoring devices and a server that can provide automated feedback to patients. The primary outcome will be hospital readmission rate during the 12 months of study enrollment. Secondary outcomes will include the total cost of hospitalizations. We will enroll patients who have been discharged from UPHS with CHF at least once in the past 12 months and randomize them to either: [1] usual care with no additional intervention; [2] the provision of wireless pill bottles and scales with daily lottery incentives to encourage daily adherence [3] the designation of a friend or family member to be a support partner for patient [4] alerts to the patient's clinician through the EMR for verified weight gains**.** To leverage access to the UPHS CHF clinic, EPIC resources for identifying eligible patients, and the Way to Health platform to launch and enroll a practical clinical trial powered to detect differences in hospital readmission rates using remote monitoring devices for automated hovering for CHF management. Primary outcome variable is the re-hospitalization rate over 12 months of enrollment. Secondary outcome variable is to assess the cost-effectiveness of the intervention relative to usual care.

2. Background

About 5.8 million Americans have CHF; 1 in 6 will develop CHF during their lifetime; and CHF is one of the most common reasons for hospital admission, hospital readmission, and a major cause of morbidity, mortality, and increasing health care costs. Among all adult patient groups, older patients with CHF have the highest rate of rehospitalization (27% within 30 days of discharge), with annual health care costs exceeding $24.3 billion. This is because patients discharged after a CHF hospital admission face many challenges, including a complex array of medications and follow-up care and the need for daily self-management, including significant lifestyle changes. Current approaches to the management of CHF show mixed elements of promise and disappointment. Complex disease management, some involving remote monitoring and some with intensive and expensive case management by nurse practitioners, have been shown in some small studies to reduce readmissions and improve survival and quality of life, but they are costly and require a workforce that is unlikely to be economically feasible to support. In addition, more recent and larger studies suggest that these approaches are ineffective. At the other extreme, trials of self-management using patients as their own workforce through intensive training demonstrate no mortality or rehospitalization benefit. These results suggest that neither conventional telemonitoring assessing symptoms and weight, nor self-management, is likely to offer promise in CHF management. A significant challenge to the effectiveness of remote monitoring programs in practice is that many patients offered such support do not participate at all and many who do participate lose interest rapidly. In our own clinics, we have found that simply providing remote monitoring devices to patients at high risk of disease exacerbations is not enough, as utilization rates of remote monitoring devices in a group asked to do this daily declined steadily over time without engagement incentives such that by 3 months patients did remote monitoring of blood pressure and blood sugars on only 50% of days. This implies that to be successful in changing behavior of patients whose behavior has likely contributed to their being high risk, remote monitoring and engagement enhancements need to be combined. There is consensus that the effective management of CHF depends on medication adherence, dietary management (particularly sodium), and weight management (to monitor fluid balance). Medication adherence and weight are much easier to monitor than food intake. Low adherence to CHF medications is associated with more admissions and higher mortality. Non-adherence rates are high,

with estimates ranging from adherence to angiotensin-converting enzyme inhibitors of only 60% at 1 year to full adherence, defined as filling enough prescriptions to have medication available each day for 1 year, as low as 10%. The data on the importance of weight management for CHF patients is compelling; often the earliest sign of pending decompensation is a gain in weight of several pounds that presages clinical deterioration, which can be avoided by early intervention. Weight monitoring adherence (odds ratio [OR] 0.42, 95% confidence interval [CI] 0.23-0.76) and diuretic self-adjustment adherence (OR 0.44, 95% CI 0.19-0.98) have been found to be associated with lower adjusted odds of CHF-related ED visits or hospitalizations. For these reasons, we will focus on adherence to both daily weights and diuretic use in this intervention. Further, this study would be the first to experimentally test the impact on CHF management of remote medication adherence and weight monitoring along with behavioral economic engagement incentives. The engagement incentives will help us attain much higher rates of ongoing participation by high-risk patients than otherwise would be likely. This approach will enable extension of clinical services outside the clinician offices utilizing an existing technology-based approach with built in automatic feedback loops (including small, but frequent incentives that have been extensively tested in other contexts) to ensure high rates of adherence, which is an approach that minimizes personnel costs relative to traditional disease management.

3. Overall Objectives

To leverage access to the UPHS CHF clinic, EPIC resources for identifying eligible patients, and the Way to Health platform to launch and enroll a practical clinical trial powered to detect differences in hospital readmission rates using remote monitoring devices for automated hovering for CHF management.

4. Aims

4.1. Primary Aims

Re-hospitalization rate over 12 months of enrollment

4.2. Secondary Aims

To assess the cost-effectiveness of the intervention relative to usual care

5. Primary Outcome Variable

The primary analysis will consist of unadjusted intent-to-treat hypothesis tests using the logrank test to compare the time to hospitalization in the two groups.

6. Secondary Outcome Variable(s)

In secondary analyses we will investigate the sensitivity to modeling assumptions using imputation models and inverse probability-weighted estimating equations and models that adjust for informative missing data.

7. Study Design

7.1. Phase

Phase III

7.2. Design

Participants in this study will be identified using Penn Data science through regular feeds sent to the research coordinators. Potentially eligible patients will be identified while they are admitted to a UPHS hospital and provided information on the study, which will either be delivered by study staff or channeled through the health care provider. Participants will either be recruited while they are in the hospital before discharge or they will receive a recruitment phone call in the few days post-discharge. During enrollment, the coordinator will read the IRB-approved consent/HIPAA script and the participant will be asked to provide verbal consent and verbal HIPAA authorization for use of their data in the study. Once a patient provides verbal consent, the coordinator will select this option on the consent/HIPAA form on the patient profile created during the enrollment call on the WTH platform. After the patient has consented to study participation and study eligibility has been confirmed, the coordinator will enter basic demographic information for the patient onto the WTH platform and will collect information on the UPHS managing physician that the patient will be following up with for CHF. Participants will also be asked for their SSN to complete a W-9 form on the patient profile on the WTH platform. Participants will then be randomized to either the intervention or usual care (250 intervention, 250 control). Control patients will be thanked for their participation, compensated $25 for enrolling and will not be contacted again. Intervention participants will (1) be given a medication adherence device for their diuretic and a scale; (2) asked to provide the coordinator with name and contact information of a family member or friend to serve as a support partner; (3) will be assigned a 2- digit number to be used as part of the lottery-based engagement incentives in which eligibility to win will be conditional on medication adherence and registering a weight measurement; and (4) will determine their preferences for WTH platform communication methods during the study. In regards to support partners, the coordinator will reach out to the family member or friend, whose contact information will be provided by the patient during the enrollment process, to ask them to serve as the patients support partner. He or she will be given the option of receiving a text message, an e-mail, or interactive voice recording (IVR) from the WTH platform. The coordinator will place the call to the potential support partner after the patient is enrolled. If the enrollment process is completed in-person, participants will be given the medication adherence device and scale and the coordinator will provide the participant with a brief tutorial on how the devices should be used, but will schedule the participant for a follow up phone call to ensure proper set up of devices in the home. If the enrollment process is completed remotely, participants will be told that they should receive the medication adherence device and scale in the next few days to the mailing address confirmed during the enrollment process. On the enrollment call, the coordinator and patient will also schedule an appointment to setup their devices. Following enrollment (in-person or remote), we will add a note to the patients electronic medical chart saying that they are an active participant in this study. We will also contact the patients managing physician to inform them of their patients enrollment in the study and to ask them which physician pool they are associated with in PennChart. During the device setup process, the coordinator will then explain to the patient that he or she will receive daily feedback on adherence to their medication and registering a weight measurement. This feedback will include information on the lottery; patients will be eligible for the daily lottery if they had adhered to their medication and if they had registered a weight measurement on the previous day. Patients will be asked whether they would like to receive the daily feedback via text message, e-mail or IVR. If a patient is non-adherent to medication and registering a weight measurement for 2 days, an automated call will be made to the patient to stress the importance of taking medication and weighing in and an automated feedback message will be sent to the patients support partner. After 3 days of non-adherence, study staff will call the patient. After 4 days of non-adherence, study staff will call the support partner. Finally, after 5 days of non-adherence, study staff will send a note to the patients managing clinician through PennChart. All participants will be instructed to call study staff for any

questions or problems related to device use. During the device setup process, the coordinator will also explain to the patient that study staff will be monitoring their weight to see if their weight rises above a clinically-significant threshold. The patient will be asked to step on the scale twice in order to confirm that the device is setup properly and to provide a baseline weight. The coordinator will also explain to the patient that their weight measurements will be sent to their managing physician via PennChart on a weekly basis. The coordinator will send a weekly report of weight measurements via Way to Health to the managing physicians in-basket in PennChart. At the end of study participation, participants will also be asked to complete a survey about their experience in the study.

7.3. Study Duration

The duration of participation for each individual participant is 12 months from date of enrollment

7.4. Facilities

This project will take place at the Leonard Davis Institute Center for Health Incentives and Behavioral Economics (LDI CHIBE) at the University of Pennsylvania (UPenn). The team includes investigators experienced in clinical medicine, health behavior interventions, clinical trials, behavioral economics, cost-effectiveness analysis, and program evaluation. Our partnership combines the resources and capabilities of a major university (the Wharton School and the Perelman School of Medicine at the University of Pennsylvania), a major health care provider (UPHS). Multiple PIs: Dr. Kevin Volpp directs the LDI CHIBE and the NIA-funded PENN-CMU Roybal P30 Center on Behavioral Economics and Health and is a Professor of Medicine at the Perelman School of Medicine (SOM) and Professor of Health Care Management at the Wharton School at UPenn. He has led numerous studies of patient financial incentives and behavioral economic interventions. David Asch, MD, MBA is Co-Project Director and is Executive Director of the Penn Medicine Center for Innovation, Professor of Health Care Management and Economics and Professor of Operations and Information Management at Wharton and Professor of Medicine at Perelman. The financial analyses will be co-led by Dr. Shivan Mehta. Statistical Analysis: Dr. Andrea Troxel (Co-I, Statistician) is Director of Biostatistics for LDI CHIBE and a Professor of Biostatistics at UPenn. She has over 15 years of experience in the design, conduct, and analysis of clinical studies, including randomized trials that involve repeated measurements. There will be a project manager and research coordinator assigned to this study to facilitate enrollment, device distribution, follow up contacts and payment distributions. This study will be supported on a secure web portal on the WTH platform, modified to the specifications of this study.

7.5. Key Inclusion Criteria

Patients will be targeted for study recruitment if they have been discharged to home within the past 30 days from a UPHS hospital with a principal diagnosis of CHF, aged 18-80 years old, and will receive follow-up in a UPHS outpatient clinic by a cardiologist or primary care physician.

7.6. Key Exclusion Criteria

Patients will be excluded from study participation if they are less than 18 years old or older than 80 years old, will not or cannot provide informed consent, have a markedly shortened life expectancy (listed for heart transplant, have ventricular assist device, are inotrope dependent, have metastatic cancer, are currently receiving palliative care/hospice, or have dementia), have end-stage renal disease, if their glomerular filtration rate is less than 25 ml/min, if they are on dialysis, or are medically unstable, or if their heart failure is managed with a CardioMEMS monitor. Patients will also be excluded from

study participation if they are receiving another remote monitoring/telemedicine intervention or if they are receiving follow-up care outside of UPHS.

8. Subject Recruitment

8.1. Target Population

Eligibility criteria: Patients with CHF who have been discharged to home within the past 30 days from a UPHS hospital with a principal diagnosis of CHF aged 18-80 years old and will receive follow-up in a UPHS outpatient clinic by a cardiologist or primary care physician. The total target enrollment will be 500 participants.

8.2. Subjects at Penn

500

8.3. Accrual

Participants in this study will be identified primarily through a Penn Data Science feed. Penn Data Science will use a special algorithm to identify CHF patients being discharged from UPHS. Potential participants will be given a study brochure while in the hospital and their contact information will be ascertained through review of their electronic medical record. If the patients on the Penn Data Science feed meet the minimum requirements for study eligibility, they will be added to the study screening database and will be approached for recruitment in the hospital. Patients who we are unable to reach in the hospital will be contacted a few days post-discharge via phone call and will be asked to participate in the study

8.4. Patient Subject Recruitment

Participants in this study will be identified primarily through a Penn Data Science feed. Penn Data Science will use a special algorithm to identify CHF patients being discharged from UPHS. A coordinator will review the list of potential participants to confirm that they meet the minimum requirements for eligibility, and if so, their contact information will be ascertained through review of their electronic medical record. Potential participants will be given a study brochure while in the hospital as they prepare to be discharged to home. Participants will be entered into the study screening database and approached prior to discharge. If the coordinator is unable to reach the participant in the hospital, they will be contacted post-discharge via phone call and will be asked to participate in the study. When patients are approached for recruitment, a coordinator will confirm their eligibility for the study and will see if they are interested in participating. If participants are interested, the coordinator will read them the consent. The coordinator will enter patient information directly into the WTH platform for the study. After enrollment is complete, the coordinator will add a note into their electronic medical record that displays their study enrollment. The coordinator will also reach out to the patients managing physician to inform them of the patients enrollment in the study and to confirm which physician pool they are associated with in PennChart.

8.5. Subject Compensation

All participants will receive a participation payment of $25 for their time and effort to enroll in the study. In addition to this, we are providing participation payment of $25 to intervention participants who complete the setup process of their medication adherence device and scale with a study coordinator. This is to reward them for the time and effort of completing device setup for use in the study. The participants will also be eligible to receive an average expected lottery payment of $1.40/day if they are adherent to their study medication and to registering a weight measurement.

9. Study Procedures

9.1. Consent Process

We are requesting a waiver of the requirement to document consent and HIPAA authorization with a signature for participants enrolled into this study since we believe that the research presents no more than minimal risk of harm to subjects and involves no procedures for which written consent is normally required outside of the research context. [45 CFR 46.117(c)(2)] Some participants will enroll in this study via a remote recruitment process, and therefore, we will read the IRB-approved Consent/HIPAA script over the phone to each participant and ask them to provide verbal consent and verbal HIPAA authorization for use of their data in the study. After a patient provides verbal consent, the coordinator will select this option on the Consent/HIPAA screen on the participants profile that was created during enrollment on the WTH platform for this study. A copy of the Consent/HIPAA document will be included in the device packet provided to them in the hospital (if enrollment occurred in the hospital) or sent to the patients home address (if enrollment was conducted remotely). Participants recruited in the hospital prior to their discharge will be asked to provide their signature for HIPAA authorization. They will also be provided a copy of the Consent/HIPAA document.

9.1.1.1. Waiver or Alteration of Informed Consent

Waiver of written documentation of informed consent: the research presents no more than minimal risk of harm to subjects and involves no procedures for which written consent is normally required outside of the research context

9.1.1.2. Minimal Risk

This research presents no more than minimal risk of harm to subjects and involves no procedures for which written consent is normally required outside of the research context.

9.1.1.3. Impact on Subject Rights and Welfare

We will be obtaining verbal consent from patients who have been provided a thorough explanation of the study and the opportunity to ask any questions about study participation. Patients will be read the entire consent and given the option to participate.

9.1.1.4. Waiver Essential to Research

All participants will be recruited through remote procedures. Patients will be contacted by phone after they are discharged for recruitment. Patients will be mailed a copy of the informed consent/HIPAA authorization for this study if they decide to enroll

9.1.1.5. Written Statement of Research

This study operates under a written statement of research.

9.2. Procedures

Participants in this study will be identified primarily through a Penn Data Science feed. Penn Data Science will use a special algorithm to identify CHF patients being discharged from UPHS. A coordinator will review the list of potential participants to confirm that they meet the minimum requirements for eligibility. Potential participants will be given a study brochure while they are still in the hospital as they prepare to be discharged to home. Participants will be entered into the study database and approached by a coordinator prior to discharge. If the coordinator is unable to reach the patient in the hospital, they will be contacted post-discharge via phone call. When patients are approached for recruitment, a coordinator will ask the patient if they are willing to answer some screening questions to confirm their eligibility. If they agree, the coordinator will read through a brief screening survey to confirm that they meet the criteria of having a CHF diagnosis, have been prescribed a diuretic, are being or have been discharged to home, and are being managed by a primary care provider or cardiologist in UPHS. If patient eligibility is confirmed, the coordinator will read the patient the consent. The coordinator will create the patients study account on the WTH platform and directly enter relevant patient information. If, after reviewing the Consent/HIPAA document, the patient wants to participate, the coordinator will indicate that the patient provided verbal consent to participate by selecting this option on the WTH platform. Patients recruited in the hospital will be asked to provide their signature for HIPAA authorization. Patients will also be asked basic demographic information and information on their UPHS managing clinician. The coordinator will also collect the patients SSN to enter into the W-9 page on the WTH platform to facilitate participant incentive payments. Participants randomized to the intervention will be given (1) a wireless medication adherence device for their diuretic and a scale; (2) asked to provide the coordinator with name and contact information of a family member or friend to serve as a support partner; (3) will be assigned a 2-digit number to be used as part of the lottery-based engagement incentives in which eligibility to win will be conditional on medication adherence and registering a weight measurement; and (4) will determine their preferences for WTH platform communication methods during the study. The coordinator will schedule an appointment with the participant to complete setup of their devices in the home once they have received them in the mail. The patients managing physician will be notified of their enrollment in the study via PennChart. During the setup process, the coordinator will provide the participant with assistance in transferring their medication into the medication adherence device and setting up their scale. The coordinator will confirm the medication and ensure that the device alarm is set to the desired medication time. After devices are setup, participants will start to receive daily messaging about their use of the medication adherence device and scale in the last 24-hour period and whether or not their study lottery number was drawn on the study lottery system. The messages will appear similar to the following: (a) Congratulations, you took your medication and registered a weight and won the lottery on [yesterday's date]. You won ($5 or $50, depending on the number of digits that were drawn for the participant). (b) You took your medication and registered a weight, but none of your lucky numbers was drawn on [yesterday's date]. Continue to take your medication and weigh-in as you did and you could win tomorrow. (c) When the lottery was run on [today's date], you had not taken your medication or registered a weight the day before. Don't miss out! You never know when your lucky numbers may be drawn, so take your medication and weigh-in every day and you may win! (d) When the lottery was run on [today's date], you had not taken your medication the day before, but you did register a weight. Don't miss out! You never know when your lucky numbers may be drawn, so take your medication every day and continue to weigh-in and you may win! (e) When the lottery was run on [today's date], you had taken your medication the day before, but you did not register a weight. Don't miss out! You never

know when your lucky numbers may be drawn, so register a weight and continue to take your medication every day and you may win! If the participant identifies a support partner and they agree to serve in this role, they will also have an account created for them on the WTH platform for this study, which will be linked to the participants WTH profile. The support partner will receive a notification if a patient does not adhere to his or her medication and to registering a weight measurement in any consecutive 48-hour period. Any interaction that the support partner has with the participant is up to their discretion and will not be tracked for study purposes. The role of the support partner is not supposed to provide trained assistance to the participant, but rather to serve the role of the providing social support around their medication adherence and weigh-ins. Both the participant and the support partner will be given the option of receiving notifications from the WTH platform via text message, e-mail message, or IVR. Further, patients weight will be monitored on a daily basis to determine if their weight rises over a specific threshold. The weight thresholds are the following: an increase of two pounds in 24 hours or five pounds in 72 hours. If a weight gain alert is produced, the coordinator will call the patient to complete an abnormal weight measurement questionnaire to validate the weight increase. If it is determined that the patient has had a true weight increase, the coordinator will send the questionnaire responses to the patient's managing physicians through Way to Health to PennChart. Way to Health will push the abnormal weight measurement questionnaire responses to the managing physician in PennChart after the coordinator determines that a true weight increase has occurred. If the coordinator cannot reach the patient on the first phone call attempt, they will call the patient again on the following day. If the patient is unresponsive again, the coordinator will send a message to the patients managing physician through PennChart to inform them that the patient has an unconfirmed weight gain. Weight alerts will be monitored every day, including weekends and holidays. If a weight alert is produced on a weekend or holiday, the coordinator will call the patient to validate the weight increase with the abnormal weight measurement questionnaire, and if completed, will send the questionnaire responses to the patients managing physician through PennChart. At the end of study participation, participants will be made aware of their last date in the program and that they will no longer have to use the medication adherence device or the scale, and that research staff will not be monitoring their devices or reaching out to them as they have during their participation. Participants will be asked to complete a survey to provide feedback on their participation in the study

10. Analysis Plan

Prior to analysis, we will produce data summaries including graphical methods to assess data quality, examine central tendencies and distributional assumptions and randomization success. The primary analysis will consist of unadjusted intent-to-treat hypothesis tests using the logrank test to compare the time to hospitalization in the two groups. We will also estimate Cox proportional hazards regression models adjusted for the stratification variable and other covariates of interest (such as patient sex, income, race, baseline ejection fraction, and quality of life), retaining these given evidence of confounding or predictive ability. We will employ a confounder selection method based on "change in estimate" criterion. We will assess interaction terms between the a priori potential effect modifiers such as income level, race, and baseline ejection fraction. All hypothesis tests will be two-sided and models will be assessed using standard diagnostic techniques.(66-68) We will use standard approaches to assess the proportional hazards assumption and include interaction terms with time if necessary. We will also fit frailty models to analyze repeated rehospitalizations within patients; these models properly adjust for the correlation of multiple events within individuals. Handling of missing data is an important issue in all RCTs. Follow-up data may be missing if participants miss the follow-up visit or withdraw. Even in subjects who are lost to follow-up, however, we will be able to capture any events that result in hospitalization, and thus will still be able to observe our primary outcome. We will compare dropout

rates by arm, will attempt to find the reasons for missing data and will compare baseline characteristics in participants with complete vs. incomplete follow-up. In secondary analyses we will investigate the sensitivity to modeling assumptions using imputation models and inverse probability-weighted estimating equations and models that adjust for informative missing data.

11. Subject Confidentiality

To ensure that patient, physician, and other informant confidentiality is preserved, individual identifiers (such as name and medical record number) are stored in a single password protected system that is accessible to study research, analysis and IT staff only. This system is hosted on site at UPenn and is protected by a secure firewall. Once a participant is in this system, they will be given a unique study identification number (ID). Any datasets and computer files that leave the firewall will be stripped of all identifiers and individuals will be referred to by their study ID. The study ID will also be used on all analytical files. Please see attached document (WTH database security grant-protocol text FINAL) for full database security details. The medication adherence device will provide adherence data from each participant. This information is transmitted via cellular signal without any subject identifiers.

11.1. Subject Privacy

Privacy refers to the person’s desire to control access of others to themselves. Privacy concerns people, whereas confidentiality concerns data. Describe the strategies to protect privacy giving consideration to the following: The degree to which privacy can be expected in the proposed research and the safeguards that will be put into place to respect those boundaries. The methods used to identify and contact potential participants. The settings in which an individual will be interacting with an investigator. The privacy guidelines developed by relevant professions, professional associations and scholarly disciplines (e.g., psychiatry, genetic counseling, oral history, anthropology, psychology). At the time that UPenn study staff receives patient data, they will upload the patient data into the secure, web-based database (REDCap) and a study ID number will be generated for each patient. A link between the study ID number and the patient PHI will need to be maintained to ensure that the study staff can track recruitment efforts to potential participants and to avoid contacting any patients who have previously declined to participate. To ensure that patient confidentiality is preserved, individual identifiers (such as name) are stored in a single password protected system that is accessible to study research, analysis and IT staff only. This system is hosted on site at UPenn and is protected by a secure firewall. Once a participant is in this system, they will be given a unique study ID number. Any datasets and computer files that leave the firewall will be stripped of all identifiers besides the study ID and individuals will be referred to by their study ID only. The study ID will also be used on all analytical files. REDCap is a secure web application for building and managing online surveys and databases. The institution installing REDCap will store all data captured in REDCap on its own servers. Therefore, all project data is stored and hosted at the local institution (UPenn) and no project data is ever transmitted at any time by REDCap from this institution to another institution or organization. Privacy of all study data will be maintained by restricting access to the identifiable information only to approved study staff who have received subject confidentiality and privacy training. Study coordinators will access patient contact information from the database to conduct recruitment phone calls. The study coordinator will review the consent script, which will include a description of the voluntary nature of participation, the study procedures, risks and potential benefits in detail. Participants will be told that all information will be kept strictly confidential, except as required by law. Subjects will be provided a copy of the consent document. All efforts will be made by study staff to ensure subject privacy. Enrollment will be conducted by the study coordinators who will enter patient information directly into the WTH platform once a participant has consented to participate. This

database is hosted on a secure server as detailed in the subject confidentiality section. Study coordinators may have to contact patients in the intervention and their support partners during the course of the study and will use the WTH database to access contact information to facilitate this contact. If the rate of non-adherence to medication and to weighing-in, as recorded by the medication adherence device and scale, rises above a certain threshold, the study team may also contact the participant's managing physician. If a participant's weight rises above a certain threshold across a specific time period, the study team may contact the participants managing physician to notify them. This will be explained to the participant in the consent process and when the details of study participation are explained by the coordinator. PHI will not be shared with anyone outside the parameters of the study as detailed in the Consent/HIPAA process.

11.2. Data Disclosure

The following entities, aside from members of the research team, may receive PHI for this research study: CleverCap; The Office of Human Research Protections and the University of Pennsylvania; federal and state agencies (for example, the National Institutes of Health); and, other domestic or foreign government bodies if required by law and/or necessary for oversight purposes

11.3. Data confidentiality

The following methods will be employed to protect patient PHI for this research study:

x Paper-based records will be kept in a secure location and only be accessible to personnel involved in the study.

x Computer-based files will only be made available to personnel involved in the study through the use of access privileges and passwords.

x Prior to access to any study-related information, personnel will be required to sign statements agreeing to protect the security and confidentiality of identifiable information.

x Wherever feasible, identifiers will be removed from study-related information. A Certificate of Confidentiality will be obtained, because the research could place the subject at risk of criminal or civil liability or cause damage to the subject's financial standing, employability, or liability.

x A waiver of documentation of consent is being requested, because the only link between the subject and the study would be the consent document and the primary risk is a breach of confidentiality. (This is not an option for FDA-regulated research.)

x Precautions are in place to ensure the data is secure by using passwords and encryption, because the research involves web-based surveys.

12. Consent Process Overview

We are requesting a waiver of the requirement to document consent and HIPAA authorization with a signature for participants enrolled into this study since we believe that the research presents no more than minimal risk of harm to subjects and involves no procedures for which written consent is normally

required outside of the research context. [45 CFR 46.117(c)(2)] Some participants will enroll in this study via a remote recruitment process, and therefore, we will read the IRB-approved Consent/HIPAA script over the phone to each participant and ask them to provide verbal consent and verbal HIPAA authorization for use of their data in the study. After a patient provides verbal consent, the coordinator will select this option on the Consent/HIPAA screen on the participants profile that was created during enrollment on the WTH platform for this study. A copy of the Consent/HIPAA document will be included in the device packet provided to them in the hospital (if enrollment occurred in the hospital) or sent to the patients home address (if enrollment was conducted remotely). Participants recruited in the hospital prior to their discharge will be asked to provide their signature for HIPAA authorization. They will also be provided a copy of the Consent/HIPAA document.

12.1. Potential Study Risks

As this study does not involve any medical decision making and only observes the use of social behavioral approaches to encouraging patients to use evidence-based treatments that their providers have prescribed them following their CHF diagnosis, we consider this study to be minimal risk. The primary risk would be from a breach of confidentiality involving electronic medical record reviews and monitoring of medication adherence with a medication adherence device and monitoring of weights from an electronic scale, which will be maintained on the WTH platform. This risk has been mitigated by extensive privacy protection protocols, a highly secure data storage system, and a plan to remove identifiers from the data wherever possible. In addition, all study personnel will be held to high standards of upholding confidentiality and safeguarding patient privacy

12.2. Potential Study Benefits

This study is to test a new approach to chronic disease management that combines remote monitoring devices and behavioral economic engagement incentives to reduce rehospitalization rates among patients with CHF. The immediate benefits of this study for participants may include improvement for management of CHF, which may lower their risk for future morbidity and death, improved quality of life, and reduced medical care costs. Patients will be randomized into either an intervention group that receives the medication adherence device and scale or a control group that receives usual care. The potential public health impact of a successful intervention to improve management of CHF is enormous and could reduce the number of deaths in the United States substantially each year. The benefits of this research to the participants studied, and to society at large, far surpass the minimal risks.

12.3. Alternatives to Participation

Patients are free to decline participation in this study. If they decline to participate, they will receive no reduction in the usual care received from their health care providers for this condition.

12.4. Data and Safety Monitoring

While we consider this study to present minimal risk to participants, we have established a Data Safety Monitoring Board of experts in statistics and congestive heart failure to monitor adverse events that may occur among our study participants. The DSMB will be convened every six months (or more often if needed) and will review reports of discharge data for adverse events and assess for safety. Adverse events may also be self-reported by participants. This information will be reviewed by a physician on our study team and will be reported to the IRB in according to all reporting regulations. The following

documents are currently attached to this item: There are no documents attached for this item. Risk / Benefit Assessment Current limitations to managing CHF are a major public health problem with few scalable, cost-effective solutions. This study will test a novel behavioral economic intervention. We believe that this approach will provide the research and public health communities with important information that can lead to broad generalizability in treating people with CHF at risk for hospitalization and death nationally. With the large scientific and public health benefits of the knowledge gained from this study, the minimal risks to participants are reasonable in relation to the importance of the knowledge that reasonably may be expected to result.

Final Study Protocol

Table of Contents

1. Abstract 20

2. Background 20

3. Overall Objectives 21

4. Aims 21

4.1. Primary Aims 21

4.2. Secondary Aims 22

5. Primary Outcome Variable 22

6. Secondary Outcome Variable(s) 22

7. Study Design 22

7.1. Phase 22

7.2. Design 22

7.3. Study Duration 24

7.4. Facilities 24

7.5. Key Inclusion Criteria 25

7.6. Key Exclusion Criteria 25

8. Subject Recruitment 25

8.1. Target Population 25

8.2. Subjects at Penn 24

8.3. Accrual 24

8.4. Patient Subject Recruitment 25

8.5. Subject Compensation 26

9. Study Procedures 26

9.1. Consent Process 26

9.1.1. Waiver of Alerteration of Informed Consent 26

9.1.2. Minimal Risk 27

9.1.3. Impact on Subject Rights and Welfare 27
9.1.4. Waiver Essential to Research 27
9.1.5. Written Statement of Research 27
9.2. Procedures 27
10. Analysis Plan 30
11. Subject Confidentiality 31
11.1. Subject Privacy 32
11.2. Data Disclosure 33
11.3. Data confidentiality 33
12. Consent Process Overview 33
12.1. Potential Study Risks 34
12.2. Potential Study Benefits 34
12.3. Alertnatives to Participation 34
12.4. Data Safety and Monitoring 34
12.4.1. Data Safety and Monitoring Plan........34
12.4.1.1. Study Background and Significance........34
12.4.1.2. *Potential Risks and Benefit for Study Participants*........35
12.4.1.2.1. Discussion of Potential Risks........36
12.4.1.2.2. Discussion of Potential Benefits........36
12.4.1.2.3. Protection against Study Risks........36
12.4.1.2.3.1. *Informed Consent Process*........36
12.4.1.2.3.2. Patient Participant Recruitment........37
12.4.1.2.3.3. Data Safety Procedures........37
12.4.1.3. Adverse Event Monitoring and Surveillance........38
12.4.1.3.1. Adverse Event Surveillance........39
12.4.1.3.1.1. Defining Adverse Events........39
12.4.1.3.1.2. Defining Serious Adverse Events........39

12.4.1.3.1.3. Periodic Surveillance of Adverse Events……………………………….40
12.4.1.3.1.4. Adverse Event Reporting…………………………………………………….40
12.4.1.3.1.5. Expected Events…………………………………………………………………….40
12.4.1.4. Data Safety and Monitoring Board (DSMB)…………………………………………………*.40*
12.4.1.4.1. DSMB Responsibilities………………………………………………………………………….40
12.4.1.4.2. DSMB Membership and Affiliation……………………………………………………..41
12.4.1.4.3. Board Process…………………………………………………………………………………….41
12.4.1.4.4. *Meeting Format*……………………………………………………………………………………42
12.4.1.4.5. Meeting Materials……………………………………………………………………………….42
12.4.1.4.6. Reports from the DSMB………………………………………………………………………..43
12.4.1.4.7. *Confidentiality*……………………………………………………………………………………..43

1. Abstract

Using a 2-arm, randomized, controlled trial (RCT) among CHF patients at the University of Pennsylvania Health System (UPHS), we propose to test the effectiveness of applying automated hovering to improve outcomes among CHF patients at high risk of readmission. To conduct this trial we will leverage the Penn Data Store, an electronic repository that can be used to identify study cohorts, and the NIH-funded Way to Health platform, which provides linkages between home-based remote monitoring devices and a server that can provide automated feedback to patients. The primary outcome will be hospital readmission rate during the 12 months of study enrollment. Secondary outcomes will include the total cost of hospitalizations. We will enroll patients who have been discharged from UPHS with CHF at least once in the past 12 months and randomize them to either: [1] usual care with no additional intervention; [2] the provision of wireless pill bottles and scales with daily lottery incentives to encourage daily adherence [3] the designation of a friend or family member to be a support partner for patient [4] alerts to the patient's clinician through the EMR for verified weight gains. The overall objective is to leverage access to the UPHS CHF clinic, EPIC resources for identifying eligible patients, and the Way to Health platform to launch and enroll a practical clinical trial powered to detect differences in hospital readmission rates using remote monitoring devices for automated hovering for CHF management. Primary outcome variable(s) include re-hospitalization rate over 12 months of enrollment. Secondary outcome variable(s) include: 1) To assess the cost-effectiveness of the intervention relative to usual care 2) assess cause-specific rehospitalizations.

2. Background

About 5.8 million Americans have CHF; 1 in 6 will develop CHF during their lifetime; and CHF is one of the most common reasons for hospital admission, hospital readmission, and a major cause of morbidity, mortality, and increasing health care costs. Among all adult patient groups, older patients with CHF have the highest rate of rehospitalization (27% within 30 days of discharge), with annual health care costs exceeding $24.3 billion. This is because patients discharged after a CHF hospital admission face many challenges, including a complex array of medications and follow-up care and the need for daily self-management, including significant lifestyle changes. Current approaches to the management of CHF show mixed elements of promise and disappointment. Complex disease management, some involving remote monitoring and some with intensive and expensive case management by nurse practitioners, have been shown in some small studies to reduce readmissions and improve survival and quality of life, but they are costly and require a workforce that is unlikely to be economically feasible to support. In addition, more recent and larger studies suggest that these approaches are ineffective. At the other extreme, trials of self-management using patients as their own workforce through intensive training demonstrate no mortality or rehospitalization benefit. These results suggest that neither conventional telemonitoring assessing symptoms and weight, nor self-management, is likely to offer promise in CHF management. A significant challenge to the effectiveness of remote monitoring programs in practice is that many patients offered such support do not participate at all and many who do participate lose interest rapidly. In our own clinics, we have found that simply providing remote monitoring devices to patients at high risk of disease exacerbations is not enough, as utilization rates of remote monitoring devices in a group asked to do this daily declined steadily over time without engagement incentives such that by 3 months patients did remote monitoring of blood pressure and blood sugars on only 50% of days. This implies that to be successful in changing behavior of patients whose behavior has likely contributed to their being high risk, remote monitoring and engagement enhancements need to be combined. There is consensus that the effective management of CHF depends on medication adherence, dietary management (particularly sodium), and weight management (to monitor fluid balance). Medication adherence and weight are much easier to monitor than food intake. Low adherence to CHF

medications is associated with more admissions and higher mortality. Non-adherence rates are high, with estimates ranging from adherence to angiotensin-converting enzyme inhibitors of only 60% at 1 year to full adherence, defined as filling enough prescriptions to have medication available each day for 1 year, as low as 10%. The data on the importance of weight management for CHF patients is compelling; often the earliest sign of pending decompensation is a gain in weight of several pounds that presages clinical deterioration, which can be avoided by early intervention. Weight monitoring adherence (odds ratio [OR] 0.42, 95% confidence interval [CI] 0.23-0.76) and diuretic self-adjustment adherence (OR 0.44, 95% CI 0.19-0.98) have been found to be associated with lower adjusted odds of CHF-related ED visits or hospitalizations. For these reasons, we will focus on adherence to both daily weights and diuretic use in this intervention. Further, this study would be the first to experimentally test the impact on CHF management of remote medication adherence and weight monitoring along with behavioral economic engagement incentives. The engagement incentives will help us attain much higher rates of ongoing participation by high-risk patients than otherwise would be likely. This approach will enable extension of clinical services outside the clinician offices utilizing an existing technology-based approach with built in automatic feedback loops (including small, but frequent incentives that have been extensively tested in other contexts) to ensure high rates of adherence, which is an approach that minimizes personnel costs relative to traditional disease management.

3. Overall Objectives

To leverage access to the UPHS CHF clinic, EPIC resources for identifying eligible patients, and the Way to Health platform to launch and enroll a practical clinical trial powered to detect differences in hospital readmission rates using remote monitoring devices for automated hovering for CHF management.

4. *Aims*

4.1. Primary Aims

Re-hospitalization rate over 12 months of enrollment

4.2. Secondary Aims

1. To assess the cost-effectiveness of the intervention relative to usual care 2. assess cause-specific rehospitalizations

5. Primary Outcome Variable

The primary comparison will be the times to events compared between the intervention arm and the usual care arm; that is, the primary hypothesis is whether a series of interventions, adapted to the behavior and outcomes of each particular patient, will reduce readmission rates compared to usual care.

6. Secondary Outcome Variable(s)

In secondary analyses the comparison will also be times to events compared between the intervention and the usual care arm for cause-specific rehospitalizations. Cost-effectiveness ratios will be calculated as the difference in costs divided by the difference in rehospitalization rate calculated under Specific Aim 3 for the “within-trial” analysis.

7. Study Design

7.1. Phase

Phase III

7.2. Design

Participants in this study will be identified using Penn Data science through regular feeds sent to the research coordinators. Potentially eligible patients will be identified while they are discharged from a UPHS hospital and provided information on the study, which will either be delivered by study staff or channeled through the health care provider. Participants will be recruited after discharge by phone call in the few days post-discharge. During enrollment, the coordinator will read the IRB-approved consent/ HIPAA script and the participant will be asked to provide verbal consent and verbal HIPAA authorization for use of their data in the study. Once a patient provides verbal consent, the coordinator will select this option on the consent/HIPAA form on the patient profile created during the enrollment call on the WTH platform. After the patient has consented to study participation and study eligibility has been confirmed, the coordinator will enter basic demographic information for the patient onto the WTH platform and will collect information on the UPHS managing physician that the patient will be following up with for CHF. Participants will also be asked for their SSN to complete a W-9 form on the patient profile on the WTH platform. Participants will then be randomized to either the intervention or usual care (283 intervention, 283 control). Control patients will be thanked for their participation, compensated $25 for enrolling and will not be contacted again. Intervention participants will (1) be given a medication adherence device for their diuretic and a scale; (2) asked to provide the coordinator with name and contact information of a family member or friend to serve as a support partner; (3) will be assigned a 2-digit number to be used as part of the lottery-based engagement incentives in which eligibility to win will be conditional on medication adherence and registering a weight measurement; and (4) will determine their preferences for WTH platform communication methods during the study. In regards to support partners, the coordinator will reach out to the family member or friend, whose contact information will be provided by the patient during the enrollment process, to ask them to serve as the patients support partner. He or she will be given the option of receiving a text message, an e-mail, or interactive voice recording (IVR) from the WTH platform. The coordinator will place the call to the potential support partner after the patient is enrolled. Participants will be sent the medication adherence device and scale and the coordinator will provide the participant with a brief tutorial on how the devices should be used, and will be available for follow up phone scall to ensure proper set up of devices in the home. Participants will be told that they should receive the medication adherence device and scale in the next few days to the mailing address confirmed during the enrollment process. On the enrollment call, the coordinator and patient will also schedule an appointment to setup their devices. Following enrollment, we will add a note to the patients electronic medical chart saying that they are an active participant in this study. We will also contact the patients managing physician through PennChart and by email to inform them of their patients enrollment in the study. During the device setup process, the coordinator will then explain to the patient that he or she will receive daily feedback on adherence to their medication and registering a weight measurement. This feedback will include information on the lottery; patients will be eligible for the daily lottery if they had adhered to their medication and if they had registered a weight measurement on the previous day. Patients will be asked whether they would like to receive the daily feedback via text message, e-mail or IVR. If a patient is non-adherent to medication and registering a weight measurement for 2 days, an automated call will be made to the patient to stress the importance of taking medication and weighing in and an automated feedback message will be sent to the patients support partner. On 3rd day of non-adherence, study staff will call the patient. On 4th day of non-adherence, study staff will call the support partner. Finally, on 5th day of non-adherence, study staff will send a note to the patients managing clinician through PennChart. All participants will be instructed to call study staff for any questions or problems related to device use.

During the device setup process, the coordinator will also explain to the patient that study staff will be monitoring their weight to see if their weight rises above a clinically-significant threshold. The patient will be asked to step on the scale twice in order to confirm that the device is setup properly and to provide a baseline weight. The coordinator will also explain to the patient that their weight measurements will be sent to their managing physician via PennChart on a weekly basis. The coordinator will send a weekly report of weight measurements via Way to Health to the managing physicians in the patient reported flowsheet. Participants in the intervention will also be sent inspirational/motivational messages at random time points that encourage healthy behaviors for congestive heart failure. At the end of study participation, some participants will also be asked to complete an interview about their experience in the study. This project will consist of semi-structured telephone interviews with patients who participated in the intervention arm of the CHF Empower project. We will identify study participants who graduated from the program in the past four months and represented one of four categories noted in the table below. We will identify patients with high rates of adherence (90%) and low rates of adherence (or equal to 60%) as well as patients enrolled in the intervention group with any readmissions or no readmissions. Patients from each of the categories noted below will be targeted for interviews. We anticipate these numbers will double by the end of the study period as we recruit graduates prospectively. Research coordinators will conduct semi-structured telephone interviews with approximately 7-10 individuals from each group. Research coordinators will call the identified individuals and ask for verbal consent to a 45-60 minute interview. If unable to reach by phone, the coordinators will send them a letter with information on how to contact the study to complete the phone interview. Participants will receive a $50 incentive for their participation in the interview. Attached is the interview script with themes to be discussed with interview participants as well as sample questions. The questions noted below are a guide; however, interviewers are expected to probe as appropriate. All interviews will be tape recorded, and ADA Transcription service will transcribe them. The Senior Qualitative Research Scientist will assist with initial coding and oversee the development of a codebook. Two research coordinators will code each of the interviews. Codes will be compared at the completion of each set of coding, and any disparities in coding will be discussed and adjudicated by the Research Scientist. In addition, at the end of study participation, some providers will also be asked to complete an interview about their experience in the study. Providers and staff dyads (a cardiologist and their nurse, for example) will be selected based on the number of weight gain alerts they received for their patients in 2019. Providers will be categorized by the number of alerts they received (total in 2019), and then the top 2 quartiles will be eligible for contact. Research coordinators will conduct semi-structured telephone interviews with approximately 15 physicians and 15 nurses. Providers will receive an email from the PI asking them to participate. Research coordinators will follow up to schedule interviews. The identified individuals will be asked for verbal consent to a 15-30 minute interview. The attached interview guide details themes to be discussed with interview participants (providers) as well as sample questions. The questions are a guide; however, interviewers are expected to probe as appropriate. Participants (providers) will be offered a $25 Amazon gift card for their participation. All interviews will be tape recorded and ADA Transcription Service will transcribe them. The Senior Qualitative Research Scientist will assist with initial coding and oversee the development of a codebook. Two research coordinators will code each of the interviews. Codes will be compared at the completion of each set of coding, and any disparities in coding will be discussed and adjudicated by the Research Scientist.

7.3. Study Duration

The duration of participation for each individual participant is 12 months from date of enrollment

7.4. Facilities

This project will take place at the Leonard Davis Institute Center for Health Incentives and Behavioral Economics (LDI CHIBE) at the University of Pennsylvania (UPenn). The team includes investigators experienced in clinical medicine, health behavior interventions, clinical trials, behavioral economics, cost-effectiveness analysis, and program evaluation. Our partnership combines the resources and capabilities of a major university (the Wharton School and the Perelman School of Medicine at the University of Pennsylvania), a major health care provider (UPHS). Multiple PIs: Dr. Kevin Volpp directs the LDI CHIBE and the NIA-funded PENN-CMU Roybal P30 Center on Behavioral Economics and Health and is a Professor of Medicine at the Perelman School of Medicine (SOM) and Professor of Health Care Management at the Wharton School at UPenn. He has led numerous studies of patient financial incentives and behavioral economic interventions. David Asch, MD, MBA is Co-Project Director and is Executive Director of the Penn Medicine Center for Innovation, Professor of Health Care Management and Economics and Professor of Operations and Information Management at Wharton and Professor of Medicine at Perelman. The financial analyses will be co-led by Dr. Shivan Mehta. Statistical Analysis: Dr. Andrea Troxel (Co-I, Statistician) is Director of Biostatistics for LDI CHIBE and a Professor of Biostatistics at UPenn. She has over 15 years of experience in the design, conduct, and analysis of clinical studies, including randomized trials that involve repeated measurements. There will be a project manager and research coordinator assigned to this study to facilitate enrollment, device distribution, follow up contacts and payment distributions. This study will be supported on a secure web portal on the WTH platform, modified to the specifications of this study.

7.5. Key Inclusion Criteria

Patients will be targeted for study recruitment if they have been discharged to home within the past 30 days from a UPHS hospital with a principal diagnosis of CHF, aged 18-80 years old, and will receive follow-up in a UPHS outpatient clinic by a cardiologist or primary care physician.

7.6. Key Exclusion Criteria

Patients will be excluded from study participation if they are less than 18 years old or older than 80 years old, will not or cannot provide informed consent, have a markedly shortened life expectancy (either listed, have history of or are being evaluated for heart transplant, listed for or being evaluated for a ventricular assist device, are inotrope dependent, have metastatic cancer, are currently receiving palliative care/hospice), have end-stage renal disease, if their glomerular filtration rate is less than 25 ml/min, if they are on dialysis, or are medically unstable, or if their heart failure is managed with a CardioMEMS monitor. Patients will also be excluded from study participation if they are receiving another remote monitoring/telemedicine intervention or if they are receiving follow-up care outside of UPHS. They are also ineligible if they participated in the CHF pilot study. Patients will be excluded if they have a current cognitive or psychiatric condition to be defined as: psychosis schizophrenia/ schizophreniform disorder, schizoaffective disorder; severe cognitive impairment (traumatic brain injury); suicidal ideation/attempt; mania (separate from psychosis, but still affecting ability to consent/ participate); substance abuse; dementia.

8. Subject Recruitment

8.1. Target Population

Eligibility criteria: Patients with CHF who have been discharged to home within the past 30 days from a UPHS hospital with a principal diagnosis of CHF aged 18-80 years old and will receive follow-up in a

UPHS outpatient clinic by a cardiologist or primary care physician. The total target enrollment will be 566 participants.

8.2. Subjects at Penn

566

8.3. Accrual

Participants in this study will be identified primarily through a Penn Data Science feed. Penn Data Science will use a special algorithm to identify CHF patients being discharged from UPHS. Potential participants will be called after discharge and sent a study brochure if requested. Their contact information will be ascertained through review of their electronic medical record. If the patients on the Penn Data Science feed meet the requirements for study eligibility, they will be added to the study screening database and will called after discharge and asked to participate.

8.4. Patient Subject Recruitment

Participants in this study will be identified primarily through a Penn Data Science feed. Penn Data Science will use a special algorithm to identify CHF patients being discharged from UPHS. A coordinator will review the list of potential participants to confirm that they meet the requirements for eligibility, and if so, their contact information will be ascertained through review of their electronic medical record. Potential participants will be given a study brochure while in the hospital as they prepare to be discharged to home. Participants will be entered into the study screening database and called after discharge and will be asked to participate in the study. On the phone, the coordinator will confirm their eligibility for the study and will see if they are interested in participating. If participants are interested, the coordinator will read them the consent. The coordinator will enter patient information directly into the WTH platform for the study. After enrollment is complete, the coordinator will add a note into their electronic medical record that displays their study enrollment. The coordinator will also reach out to the patients managing physician via PennChart and email to inform them of the patients' enrollment in the study. A copy of the consent will be mailed to the participant.

8.5. Subject Compensation

All participants will receive a participation payment of $25 for their time and effort to enroll in the study. In addition to this, we are providing participation payment of $25 to intervention participants who complete the setup process of their medication adherence device and scale with a study coordinator. This is to reward them for the time and effort of completing device setup for use in the study. The participants will also be eligible to receive an average expected lottery payment of $1.40/day if they are adherent to their study medication and to registering a weight measurement. Participants will receive a $50 incentive for their participation in the interview. Providers will receive a $25 Amazon gift card for their participation in the interview.

9. Study Procedures

9.1. Consent Process

We are requesting a waiver of the requirement to document consent and HIPAA authorization with a signature for participants enrolled into this study since we believe that the research presents no more than minimal risk of harm to subjects and involves no procedures for which written consent is normally required outside of the research context. [45 CFR 46.117(c)(2)] Participants will enroll in this study via a

remote recruitment process, and therefore, we will read the IRB-approved Consent/HIPAA script over the phone to each participant and ask them to provide verbal consent and verbal HIPAA authorization for use of their data in the study. After a patient provides verbal consent, the coordinator will select this option on the Consent/HIPAA screen on the participants' profile that was created during enrollment on the WTH platform for this study. A copy of the Consent/HIPAA document will be included in the device packet provided to them by mail to the patients' home address.

9.1.1.1. Waiver or Alteration of Informed Consent

Waiver of written documentation of informed consent: the research presents no more than minimal risk of harm to subjects and involves no procedures for which written consent is normally required outside of the research context

9.1.1.2. Minimal Risk

This research presents no more than minimal risk of harm to subjects and involves no procedures for which written consent is normally required outside of the research context.

9.1.1.3. Impact on Subject Rights and Welfare

We will be obtaining verbal consent from patients who have been provided a thorough explanation of the study and the opportunity to ask any questions about study participation. Patients will be read the entire consent and given the option to participate.

9.1.1.4. Waiver Essential to Research

All participants will be recruited through remote procedures. Patients will be contacted by phone after they are discharged for recruitment. Patients will be mailed a copy of the informed consent/HIPAA authorization for this study if they decide to enroll

9.1.1.5. Written Statement of Research

This study operates under a written statement of research.

9.2. Procedures

Participants in this study will be identified primarily through a Penn Data Science feed. Penn Data Science will use a special algorithm to identify CHF patients being discharged from UPHS. A coordinator will review the list of potential participants to confirm that they meet the requirements for eligibility, and if so, their contact information will be ascertained through review of their electronic medical record. Participants will be entered into the study screening database and called after discharge and will be asked to participate in the study. On the phone, the coordinator will confirm their eligibility for the study and will see if they are interested in participating. If participants are interested, the coordinator will read them the consent. The coordinator will enter patient information directly into the WTH platform for the study. After enrollment is complete, the coordinator will add a note into their electronic medical record that displays their study enrollment. The coordinator will also reach out to the patients managing physician via PennChart and email to inform them of the patients enrollment in the study. A copy of the consent will be mailed to the participant. When patients are called for recruitment, a coordinator will ask the patient if they are willing to answer some screening questions to confirm their eligibility. If they agree, the coordinator will read through a brief screening survey to confirm that they meet the criteria of having a CHF diagnosis, have been prescribed a diuretic, are being or have been discharged to home,

and are being managed by a primary care provider or cardiologist in UPHS. If patient eligibility is confirmed, the coordinator will read the patient the consent. The coordinator will create the patients study account on the WTH platform and directly enter relevant patient information. If, after reviewing the Consent/HIPAA document, the patient wants to participate, the coordinator will indicate that the patient provided verbal consent to participate by selecting this option on the WTH platform. Patients will also be asked basic demographic information and information on their UPHS managing clinician. The coordinator will also collect the patients SSN to enter into the W-9 page on the WTH platform to facilitate participant incentive payments. Participants randomized to the intervention will be given (1) a wireless medication adherence device for their diuretic and a scale; (2) asked to provide the coordinator with name and contact information of a family member or friend to serve as a support partner; (3) will be assigned a 2-digit number to be used as part of the lottery-based engagement incentives in which eligibility to win will be conditional on medication adherence and registering a weight measurement; and (4) will determine their preferences for WTH platform communication methods during the study. The coordinator will schedule an appointment with the participant to complete setup of their devices in the home once they have received them in the mail. The patients managing physician will be notified of their enrollment in the study via PennChart. During the setup process, the coordinator will provide the participant with assistance in transferring their medication into the medication adherence device and setting up their scale. The coordinator will confirm the medication and ensure that the device alarm is set to the desired medication time. After devices are setup, participants will start to receive daily messaging about their use of the medication adherence device and scale in the last 24-hour period and whether or not their study lottery number was drawn on the study lottery system. The messages will appear similar to the following: (a) Congratulations, you took your medication and registered a weight and won the lottery on [yesterday's date]. You won ($5 or $50, depending on the number of digits that were drawn for the participant). (b) You took your medication and registered a weight, but none of your lucky numbers was drawn on [yesterday's date]. Continue to take your medication and weigh-in as you did and you could win tomorrow. (c) When the lottery was run on [today's date], you had not taken your medication or registered a weight the day before. Don't miss out! You never know when your lucky numbers may be drawn, so take your medication and weigh-in every day and you may win! (d) When the lottery was run on [today's date], you had not taken your medication the day before, but you did register a weight. Don't miss out! You never know when your lucky numbers may be drawn, so take your medication every day and continue to weigh-in and you may win! (e) When the lottery was run on [today's date], you had taken your medication the day before, but you did not register a weight. Don't miss out! You never know when your lucky numbers may be drawn, so register a weight and continue to take your medication every day and you may win! If the participant identifies a support partner and they agree to serve in this role, they will also have an account created for them on the WTH platform for this study, which will be linked to the participants WTH profile. The support partner will receive a notification if a patient does not adhere to his or her medication and to registering a weight measurement in any consecutive 48-hour period. Any interaction that the support partner has with the participant is up to their discretion and will not be tracked for study purposes. The role of the support partner is not supposed to provide trained assistance to the participant, but rather to serve the role of the providing social support around their medication adherence and weigh-ins. Both the participant and the support partner will be given the option of receiving notifications from the WTH platform via text message, e-mail message, or IVR. Further, patients weight will be monitored on a daily basis to determine if their weight rises over a specific threshold. The weight thresholds are the following: an increase of two pounds in 24 hours or five pounds in 72 hours. If a weight gain alert is produced, the coordinator will call the patient to complete an abnormal weight measurement questionnaire to validate the weight increase. If it is determined that the patient has had a true weight increase, the coordinator will send the questionnaire responses to the patient's managing physicians through Way to

Health to PennChart. Way to Health will push the abnormal weight measurement questionnaire responses to the managing physician in PennChart after the coordinator determines that a true weight increase has occurred. If the coordinator cannot reach the patient on the first phone call attempt, they will call the patient again on the following day. If the patient is unresponsive again, the coordinator will send a message to the patients managing physician through PennChart to inform them that the patient has an unconfirmed weight gain. Weight alerts will be monitored every day, including weekends and holidays. If a weight alert is produced on a weekend or holiday, the coordinator will call the patient to validate the weight increase with the abnormal weight measurement questionnaire, and if completed, will send the questionnaire responses to the patients managing physician through PennChart. Participants will also receive motivational/ inspirational messages on random dates throughout the 12 months. These messages are delivered automatically through the Way to Health platform via the participant's choice of communication (text, email, IVR). Additional extended non-adherence letters that will be sent to intervention patients and their providers at specified time points if a) they were never set up on their devices after multiple attempts to reach them by study staff or b) if they were using their devices and then stopped and study staff has been unable to reach them after multiple attempts. The patient letter for extended nonadherence will be sent at 2 weeks, 3 months and 6 months. The patient letter for never set up on devices will be sent out 4 weeks after non being able to reach patient. The provider letter for extended nonadherence will be sent after 3 weeks, 3 months and 6 months of being unable to reach the patient. The provider letter for never set up on devices will be sent out after 5 weeks of being unable to reach the patient. These additional attempts are being implemented because when intervention patients do not use their devices, we are unable to monitor their weights and medication adherence, and cannot route a result to their care teams that alert them of any clinically significant weight gains. At the end of study participation, participants will be made aware of their last date in the program and that they will no longer have to use the medication adherence device or the scale, and that research staff will not be monitoring their devices or reaching out to them as they have during their participation. Participants will be asked to complete a survey to provide feedback on their participation in the study. Patients randomized to the control group will be sent a hard copy of the informed consent and letter verifying enrollment with study contact information. For the qualitative interviews, Clinical Research Coordinators will contact selected participants and providers via telephone to request their participation in a semi-structured interview. Those participants who are not reached by phone will be sent a follow up letter with contact information for how to complete the phone interview. Providers will be sent an email to invite them to participate, followed up by a phone call. Consent will be requested on the phone, and after verbal consent is granted, the interview will begin. Interviews will take approximately 45-60 minutes for participants and 15-30 minutes for providers. Providers will receive an email from the PI asking them to participate. Research coordinators will follow up to schedule interviews with providers. Those identified individuals will be asked for verbal consent to a 15-30 minute interview. All interviews will be tape recorded, and ADA Transcription service will transcribe them. The Senior Qualitative Research Scientist will assist with initial coding and oversee the development of codebooks. Two research coordinators will code each of the interviews. Codes will be compared at the completion of each set of coding, and any disparities in coding will be discussed and adjudicated by the Research Scientist.

10. Analysis Plan

Our primary outcome is time to readmission for any cause. The primary comparison will be the times to events compared between the intervention arm and the usual care arm; that is, the primary hypothesis is whether a series of interventions, adapted to the behavior and outcomes of each particular patient, will reduce readmission rates compared to usual care. We expect the 1-year event rate to be 47% in the

usual care arm based on data obtained on our target population at UPHS. We wish to have 80% power to detect an absolute decrease in this rate of about 10% in the intervention group; this corresponds to a hazard ratio of 0.73. We will accrue 566 patients over the 24-month enrollment period and follow them for an additional 12 months; with the 1:1 randomization ratio, we will have approximately 283 subjects in each arm. The primary hypothesis test will use a two-sided Type I error rate of 0.05. Note that the power calculations are based on the time to first readmission; if patients experience multiple readmissions, statistical power will be increased. Prior to analysis, we will produce data summaries including graphical methods to assess data quality, examine central tendencies and distributional assumptions and randomization success. The primary analysis will consist of an unadjusted intent-to-treat hypothesis tests using the Andersen-Gill formulation of the Cox proportional hazards model to compare the times to hospitalizations in the two groups; these models properly adjust for the correlation of multiple repeated events within individuals. We will also estimate multivariate regression models adjusted for the stratification variable and other covariates of interest (such as patient sex, income, race, baseline ejection fraction, and quality of life), retaining these given evidence of confounding or predictive ability. We will employ a confounder selection method based on "change in estimate" criterion. We will assess interaction terms between the a priori potential effect modifiers such as income level, race, and baseline ejection fraction. All hypothesis tests will be two-sided and models will be assessed using standard diagnostic techniques. We will use standard approaches to assess the modeling assumptions and include interaction terms with time if necessary. Handling of missing data is an important issue in all RCTs. Follow-up data may be missing if participants miss the follow-up visit or withdraw. Even in subjects who are lost to follow-up, we will be able to capture any events that result in hospitalization, and thus will still be able to observe our primary outcome. This includes hospital discharges from Penn Medicine as well as obtaining state-based discharge data and from Pennsylvania, New Jersey and Delaware to ensure that all readmissions that occur outside of Penn Medicine are captured. In addition, some subjects may receive a heart transplant during the course of the study. Because the disease trajectory and treatment paradigm change significantly with that event, we will censor patients at the time of transplant; the admission to conduct the transplant will count as an event, but follow-up will cease then and patients will be removed from the risk set. We will compare dropout rates by arm, will attempt to find the reasons for missing data and will compare baseline characteristics in participants with complete vs. incomplete follow-up. If enrolled intervention patients receive a heart transplant or ventricular assist device during their 12 month participation they will no longer be eligible to participate in the remote monitoring and their data will be censored from the date they receive the transplant or VAD going forward. An additional secondary endpoint will be cause-specific rehospitalizations, the rationale being that evaluating cause-specific hospital admission will allow a more complete understanding of the potential effects of the intervention, by indicating how many of the readmissions are due to what cause (e.g., heart failure, MI, stroke, other cardiovascular cause, noncardiovascular cause, etc). In secondary analyses we will investigate the sensitivity to modeling assumptions using imputation models and inverse-probability-weighted estimating equations and models that adjust for informative missing data. Patient days spent hospitalized, in rehab or at a SNF will not count towards adherence and will be removed from numerator and denominator and considered unmeasurable for adherence. For the qualitative interviews, the Research Scientist and Clinical Research Coordinators will review all data for recurring themes. As our interest is in the differences and similarities between the groups of patients noted above, we will conduct cross-case analyses to identify the factors that may be associated with patient adherence and readmission. The analysis will consist of describing factors in each of the groups and then comparing those factors across groups.

To assess the cost-effectiveness of the interventions, we will use analytic methods for economic evaluations in clinical trials. Our approach will be similar to Specific Aim 3 using cost as the outcome. We

will use generalized linear models to adjust for the stratification variable and other factors. Cost-effectiveness ratios will be calculated as the difference in costs divided by the difference in rehospitalization rate calculated under Specific Aim 3 for the "within-trial" analysis, with parametric 95% CIs for the cost per percentage point increase in adherence and acceptability curves. Standard errors and the correlation of the difference in cost and effect will be obtained using a bootstrap procedure. A further cost-effectiveness analysis from the "societal perspective" will be conducted to assess the impact of the rehospitalization rate reductions measured as cost per QALY gained. To address uncertainty in the micro simulation model, we will also conduct a probabilistic sensitivity analysis (PSA162) by defining probability distributions for the variables in the model used to calculate costs and effectiveness. We will use the results of the PSA to calculate confidence (or credible) intervals and acceptability curves

11. Subject Confidentiality

To ensure that patient, physician, and other informant confidentiality is preserved, individual identifiers (such as name and medical record number) are stored in a single password protected system that is accessible to study research, analysis and IT staff only. This system is hosted on site at UPenn and is protected by a secure firewall. Once a participant is in this system, they will be given a unique study identification number (ID). For obtaining Pennsylvania Healthcare Cost Containment Council data (PHC4), the individual study IDs will be combined with SSN and name in order be linked with state-based discharge data to ensure all hospitalizations are being captured for the primary outcome. Hospital discharge data received from PHC4 will be de-identified and not include PHI. For obtaining NJDOH state-based hospitalization data, NJDOH will send state-wide inpatient data from 2016-2020 with name, DOB, address, gender, race, and ethnicity. Our data analysts will match on these variables to the enrolled participants. For obtaining DE state-based hospitalization data, the investigators will provide study start and end dates for entire project and DE will send identifiable inpatient hospitalization data to the investigators for that time period and the investigators will link to individually enrolled patients. Please note that for assessing hospitalizations at Penn Medicine, inpatient data will be assessed using PennChart, including any CareEverywhere data that has been imported into PennChart from outside Penn Med hospitals and that is already a part of the patient's PennChart record. Any other datasets and computer files that leave the firewall will be stripped of all identifiers and individuals will be referred to by their study ID. The study ID will also be used on all analytical files. Please see attached document (W2H.Summary.of.Data.Protections) for full database security details. The medication adherence device will provide adherence data from each participant. This information is transmitted via cellular signal without any subject identifiers.

11.1. Subject Privacy

Privacy refers to the person's desire to control access of others to themselves. Privacy concerns people, whereas confidentiality concerns data. Describe the strategies to protect privacy giving consideration to the following: The degree to which privacy can be expected in the proposed research and the safeguards that will be put into place to respect those boundaries. The methods used to identify and contact potential participants. The settings in which an individual will be interacting with an investigator. The privacy guidelines developed by relevant professions, professional associations and scholarly disciplines (e.g., psychiatry, genetic counseling, oral history, anthropology, psychology). At the time that UPenn study staff receives patient data, they will upload the patient data into the secure, web-based database (REDCap) and a study ID number will be generated for each patient. A link between the study ID number and the patient PHI will need to be maintained to ensure that the study staff can track recruitment

efforts to potential participants and to avoid contacting any patients who have previously declined to participate. To ensure that patient confidentiality is preserved, individual identifiers (such as name) are stored in a single password protected system that is accessible to study research, analysis and IT staff only. This system is hosted on site at UPenn and is protected by a secure firewall. Once a participant is in this system, they will be given a unique study ID number. Any datasets and computer files that leave the firewall will be stripped of all identifiers besides the study ID and individuals will be referred to by their study ID only. The study ID will also be used on all analytical files. REDCap is a secure web application for building and managing online surveys and databases. The institution installing REDCap will store all data captured in REDCap on its own servers. Therefore, all project data is stored and hosted at the local institution (UPenn) and no project data is ever transmitted at any time by REDCap from this institution to another institution or organization. Privacy of all study data will be maintained by restricting access to the identifiable information only to approved study staff who have received subject confidentiality and privacy training. Study coordinators will access patient contact information from the database to conduct recruitment phone calls. The study coordinator will review the consent script, which will include a description of the voluntary nature of participation, the study procedures, risks and potential benefits in detail. Participants will be told that all information will be kept strictly confidential, except as required by law. Subjects will be provided a copy of the consent document. All efforts will be made by study staff to ensure subject privacy. Enrollment will be conducted by the study coordinators who will enter patient information directly into the WTH platform once a participant has consented to participate. This database is hosted on a secure server as detailed in the subject confidentiality section. Study coordinators may have to contact patients in the intervention and their support partners during the course of the study and will use the WTH database to access contact information to facilitate this contact. If the rate of non-adherence to medication and to weighing-in, as recorded by the medication adherence device and scale, rises above a certain threshold, the study team may also contact the participants' managing physician. If a participants' weight rises above a certain threshold across a specific time period, the study team may contact the participants managing physician to notify them. This will be explained to the participant in the consent process and when the details of study participation are explained by the coordinator. PHI will not be shared with anyone outside the parameters of the study as detailed in the Consent/HIPAA process.

11.2. Data Disclosure

The following entities, aside from members of the research team, may receive PHI for this research study: AdhereTech; Qualcomm/Integron The Office of Human Research Protections and the University of Pennsylvania; federal and state agencies (for example, the National Institutes of Health, the NJDOH, and the Pennsylvania Healthcare Cost Containment Council, Delaware Health and Social Services); and, other domestic or foreign government bodies if required by law and/or necessary for oversight purposes. Andrea Troxel, statistician, has left the University of Pennsylvania and moved to New York University. She will act as an adviser on this project and only have access to de-identified data.

11.3. Data confidentiality

The following methods will be employed to protect patient PHI for this research study:

x Paper-based records will be kept in a secure location and only be accessible to personnel involved in the study.

x Computer-based files will only be made available to personnel involved in the study through the use of access privileges and passwords.

x Prior to access to any study-related information, personnel will be required to sign statements agreeing to protect the security and confidentiality of identifiable information.

x Wherever feasible, identifiers will be removed from study-related information. A Certificate of Confidentiality will be obtained, because the research could place the subject at risk of criminal or civil liability or cause damage to the subject's financial standing, employability, or liability.

x A waiver of documentation of consent is being requested, because the only link between the subject and the study would be the consent document and the primary risk is a breach of confidentiality. (This is not an option for FDA-regulated research.)

x Precautions are in place to ensure the data is secure by using passwords and encryption, because the research involves web-based surveys.

12. Consent Process Overview

We are requesting a waiver of the requirement to document consent and HIPAA authorization with a signature for participants enrolled into this study since we believe that the research presents no more than minimal risk of harm to subjects and involves no procedures for which written consent is normally required outside of the research context. [45 CFR 46.117(c)(2)] Participants will enroll in this study via a remote recruitment process, and therefore, we will read the IRB-approved Consent/HIPAA script over the phone to each participant and ask them to provide verbal consent and verbal HIPAA authorization for use of their data in the study. After a patient provides verbal consent, the coordinator will select this option on the Consent/HIPAA screen on the participants' profile that was created during enrollment on the WTH platform for this study. A copy of the Consent/HIPAA document will be included in the device packet provided to them by mail to the patients' home address.

12.1. Potential Study Risks

As this study does not involve any medical decision making and only observes the use of social behavioral approaches to encouraging patients to use evidence-based treatments that their providers have prescribed them following their CHF diagnosis, we consider this study to be minimal risk. The primary risk would be from a breach of confidentiality involving electronic medical record reviews and monitoring of medication adherence with a medication adherence device and monitoring of weights from an electronic scale, which will be maintained on the WTH platform. This risk has been mitigated by extensive privacy protection protocols, a highly secure data storage system, and a plan to remove identifiers from the data wherever possible. In addition, all study personnel will be held to high standards of upholding confidentiality and safeguarding patient privacy.

12.2. Potential Study Benefits

This study is to test a new approach to chronic disease management that combines remote monitoring devices and behavioral economic engagement incentives to reduce rehospitalization rates among patients with CHF. The immediate benefits of this study for participants may include improvement for

management of CHF, which may lower their risk for future morbidity and death, improved quality of life, and reduced medical care costs. Patients will be randomized into either an intervention group that receives the medication adherence device and scale or a control group that receives usual care. The potential public health impact of a successful intervention to improve management of CHF is enormous and could reduce the number of deaths in the United States substantially each year. The benefits of this research to the participants studied, and to society at large, far surpass the minimal risks.

12.3. Alternatives to Participation

Patients are free to decline participation in this study. If they decline to participate, they will receive no reduction in the usual care received from their health care providers for this condition.

12.4. Data and Safety Monitoring

This study has been determined to present minimal risk to participants. However, we have established a Data Safety and Monitoring Board of experts in statistics and congestive heart failure to monitor adverse events that occur with study participants. Every 3 to 6 months, all readmissions information is pulled from the UPHS EMR and two residents independently adjudicate every hospitalization. If there is discordance between adjudications, our heart failure expert, Dr. Lee Goldberg, makes the final adjudication. These reports are presented to the DSMB (blinded by arm) so that they can then be assessed for safety. The DSMB report (blinded and de-identified) and DSMB assessments will be submitted to the IRB at the time of Continuing Review.

12.4.1. Data and Safety Monitoring Plan

12.4.1.1. Study Background and Significance

About 5.8 million Americans have CHF; 1 in 6 will develop CHF during their lifetime; and CHF is of the most common reasons for hospital admission, hospital readmission, and a major cause of morbidity, mortality, and increasing health care costs. Among all adult patient groups, older patients with CHF have the highest rate of rehospitalization (27% within 30 days of discharge), with annual health care costs exceeding $24.3 billion. This is because patients discharged after a CHF hospital admission face many challenges, including a complex array of medications and follow-up care and the need for daily self-management, including significant lifestyle changes. Current approaches to the management of CHF show mixed elements of promise and disappointment. Complex disease management, some involving remote monitoring and some with intensive and expensive case management by nurse practitioners, have been shown in some small studies to reduce readmissions and improve survival and quality of life, but they are costly and require a workforce that is unlikely to be economically feasible to support. In addition, more recent and larger studies suggest that these approaches are ineffective. At the other extreme, trials of self-management using patients as their own workforce through intensive training demonstrate no mortality or rehospitalization benefit. These results suggest that neither conventional telemonitoring assessing symptoms and weight, nor self-management, is likely to offer promise in CHF management. A significant challenge to the effectiveness of remote monitoring programs in practice is that many patients offered such support do not participate at all and many who do participate lose interest rapidly. In our own clinics, we have found that simply providing remote monitoring devices to patients at high risk of disease exacerbations is not enough, as utilization rates of remote monitoring devices in a group asked to do this daily declined steadily over time without engagement incentives such that by 3 months patients did remote monitoring of blood pressure and blood sugars on only 50% of days. This implies that to be successful in changing behavior of patients whose behavior has likely

contributed to their being high risk, remote monitoring and engagement enhancements need to be combined. There is consensus that the effective management of CHF depends on medication adherence, dietary management (particularly sodium), and weight management (to monitor fluid balance). Medication adherence and weight are much easier to monitor than food intake. Low adherence to CHF medications is associated with more admissions and higher mortality. Nonadherence rates are high, with estimates ranging from adherence to angiotensin-converting enzyme inhibitors of only 60% at 1 year to full adherence, defined as filling enough prescriptions to have medication available each day for 1 year, as low as 10%. The data on the importance of weight management for CHF patients is compelling; often the earliest sign of pending decompensation is a gain in weight of several pounds that presages clinical deterioration, which can be avoided by early intervention. Weight monitoring adherence (odds ratio [OR] 0.42, 95% confidence interval [CI] 0.23-0.76) and diuretic self-adjustment adherence (OR 0.44, 95% CI 0.19-0.98) have been found to be associated with lower adjusted odds of CHF-related ED visits or hospitalizations. For these reasons, we will focus on adherence to both daily weights and diuretic use in this intervention. Further, this study would be the first to experimentally test the impact on CHF management of remote medication adherence and weight monitoring along with behavioral economic engagement incentives. The engagement incentives will help us attain much higher rates of ongoing participation by high-risk patients than otherwise would be likely. This approach will enable extension of clinical services outside the clinician offices utilizing an existing technology-based approach with built in automatic feedback loops (including small, but frequent incentives that have been extensively tested in other contexts) to ensure high rates of adherence, which is an approach that minimizes personnel costs relative to traditional disease management.

12.4.1.2. Potential Risks and Benefit for Study Participants

12.4.1.2.1. Discussion of Potential Risks

As this study does not involve any medical decision making and only observes the use of social behavioral approaches to encouraging patients to use evidence-based treatments that their providers have prescribed them following their CHF diagnosis, the study has been deemed minimal risk. The primary risk would be from a breach of confidentiality involving electronic medical record reviews and monitoring of medication adherence with a medication adherence device and monitoring of weights from an electronic scale, which will be maintained on the WTH platform. This risk has been mitigated by extensive privacy protection protocols, a highly secure data storage system, and a plan to remove identifiers from the data wherever possible. In addition, all study personnel will be held to high standards of upholding confidentiality and safeguarding patient privacy.

12.4.1.2.2. Discussion of Potential Benefits

This study is to test a new approach to chronic disease management that combines remote monitoring devices and behavioral economic engagement incentives to reduce rehospitalization rates among patients with CHF. The immediate benefits of this study for participants may include improvement for management of CHF, which may lower their risk for future morbidity and death, improved quality of life, and reduced medical care costs. Patients will be randomized into either an intervention group that receives the medication adherence device and scale or a control group that receives usual care. The potential public health impact of a successful intervention to improve management of CHF is enormous and could reduce the number of deaths in the United States substantially each year. The benefits of this research to the participants studied, and to society at large, far surpass the minimal risks.

12.4.1.2.3. Protection against Study Risks

12.4.1.2.3.1. Informed Consent Process

The investigator will provide for the protection of the subjects by following all applicable regulations. The informed consent form will be submitted to the IRB for review and approval.

The following conditions apply to obtaining informed consent within this study:

- Waiver of HIPAA authorization (for telephone screening and verbal consent for University of Pennsylvania patients by authorized study staff who are on the IRB protocol and adhering to data protection procedures and guidelines.
- Waiver of documentation of consent (for eligible patients)

Before any procedures specified in this protocol are performed, a patient participant must:

- Be informed of all pertinent aspects of the study and all elements of informed consent.
- Be offered the opportunity to ask questions and time to consider the decision to participate.
- Voluntarily agree to participate in the study.
- Verbally agree to participate in the study.
- After a patient provides verbal consent, the coordinator will select this option on the Consent/HIPAA screen on the participant's profile that was created during enrollment on the Way To Health platform for this study. A copy of the Consent/HIPAA document will be sent to participant. Date and time documentation of this will be available on the Way to Health platform.

12.4.1.2.3.2. Patient Participant Recruitment

Participants in this study will be identified primarily through a Penn Data Science feed. Penn Data Science will use a special algorithm to identify CHF patients being discharged from UPHS. A coordinator will review the list of potential participants to confirm that they meet the requirements for eligibility, and if so, their contact information will be ascertained through review of their electronic medical record. Participants will be entered into the study screening database and called after discharge and will be asked to participate in the study. On the phone, the coordinator will confirm their eligibility for the study and will see if they are interested in participating. If participants are interested, the coordinator will read them the consent. The coordinator will enter patient information directly into the WTH platform for the study. After enrollment is complete, the coordinator will add a note into their electronic medical record that displays their study enrollment. The coordinator will also reach out to the patients managing physician via PennChart and email to inform them of the patients enrollment in the study. A copy of the consent will be mailed to the participant. When patients are called for recruitment, a coordinator will ask the patient if they are willing to answer some screening questions to confirm their eligibility. If they agree, the coordinator will read through a brief screening survey to confirm that they meet the criteria of having a CHF diagnosis, have been prescribed a diuretic, are being or have been discharged to home, and are being managed by a primary care provider or cardiologist in UPHS. If patient eligibility is confirmed, the coordinator will read the patient the consent. The coordinator will create the patients study account on the WTH platform and directly enter relevant patient information. If, after reviewing the Consent/HIPAA document, the patient wants to participate, the coordinator will indicate that the patient provided verbal consent to participate by selecting this option on the WTH platform. Patients will also be asked basic demographic information and information on their UPHS managing clinician. The

coordinator will also collect the patients' SSN to enter into the W-9 page on the WTH platform to facilitate participant incentive payments.

12.4.1.2.3.3. Data Safety Procedures

Privacy refers to the person's desire to control access of others to themselves. Privacy concerns people, whereas confidentiality concerns data. Describe the strategies to protect privacy giving consideration to the following: The degree to which privacy can be expected in the proposed research and the safeguards that will be put into place to respect those boundaries. The methods used to identify and contact potential participants. The settings in which an individual will be interacting with an investigator. The privacy guidelines developed by relevant professions, professional associations and scholarly disciplines (e.g., psychiatry, genetic counseling, oral history, anthropology, psychology). At the time that UPenn study staff receives patient data, they will upload the patient data into the secure, web-based database (REDCap) and a study ID number will be generated for each patient. A link between the study ID number and the patient PHI will need to be maintained to ensure that the study staff can track recruitment efforts to potential participants and to avoid contacting any patients who have previously declined to participate. To ensure that patient confidentiality is preserved, individual identifiers (such as name) are stored in a single password protected system that is accessible to study research, analysis and IT staff only. This system is hosted on site at UPenn and is protected by a secure firewall. Once a participant is in this system, they will be given a unique study ID number. Any datasets and computer files that leave the firewall will be stripped of all identifiers besides the study ID and individuals will be referred to by their study ID only. The study ID will also be used on all analytical files. REDCap is a secure web application for building and managing online surveys and databases. The institution installing REDCap will store all data captured in REDCap on its own servers. Therefore, all project data is stored and hosted at the local institution (UPenn) and no project data is ever transmitted at any time by REDCap from this institution to another institution or organization. Privacy of all study data will be maintained by restricting access to the identifiable information only to approved study staff who have received subject confidentiality and privacy training. Study coordinators will access patient contact information from the database to conduct recruitment phone calls. The study coordinator will review the consent script, which will include a description of the voluntary nature of participation, the study procedures, risks and potential benefits in detail. Participants will be told that all information will be kept strictly confidential, except as required by law. Subjects will be provided a copy of the consent document. All efforts will be made by study staff to ensure subject privacy. Enrollment will be conducted by the study coordinators who will enter patient information directly into the WTH platform once a participant has consented to participate. This database is hosted on a secure server as detailed in the subject confidentiality section. Study coordinators may have to contact patients in the intervention and their support partners during the course of the study and will use the WTH database to access contact information to facilitate this contact. If the rate of non-adherence to medication and to weighing-in, as recorded by the medication adherence device and scale, rises above a certain threshold, the study team may also contact the participant's managing physician. If a participant's weight rises above a certain threshold across a specific time period, the study team may contact the participants managing physician to notify them. This will be explained to the participant in the consent process and when the details of study participation are explained by the coordinator. PHI will not be shared with anyone outside the parameters of the study as detailed in the Consent/HIPAA process.

12.4.1.3. Adverse Event Monitoring and Surveillance

The study will monitor the medical safety of participants. One aspect of this monitoring is to evaluate potential participants at screening to determine whether it is safe for them to participate in the planned intervention.

Participants' safety will be monitored once they are enrolled in the trial. If a subject in the intervention has a medical illness in relation to study procedures (i.e. weighing themselves or opening the pill bottle), the safety of continuing or resuming participation in the study will be determined by the participant's managing clinician in conjunction with the Principal Investigators.

This study has been determined to present minimal risk to participants. However, we have established a Data Safety and Monitoring Board of experts in statistics and congestive heart failure to monitor adverse events that occur with study participants. Every 3 to 6 months, all readmissions information is pulled from the Penn Medicine EMR and two residents independently adjudicate every hospitalization. If there is discordance between adjudications, our heart failure expert, Dr. Lee Goldberg, makes the final adjudication. These reports are presented to the DSMB (blinded by arm) every quarter so that they can then be assessed for safety. The DSMB report (blinded and de-identified) and DSMB assessments will be submitted to the IRB at the time of Continuing Review.

12.4.1.3.1. Adverse Event Surveillance

12.4.1.3.1.1. Defining Adverse Events

According to the *Penn Manual for Clinical Research*, Adverse Events are events that:

- Are unanticipated ***--and--***
- Suggest that participants or others are at greater risk of harm than was previously known or recognized.

This definition specifically includes the following:

- Information that indicates a change to the risks or potential benefits of the research, in terms of severity or frequency.
- Breach of confidentiality.
- Incarceration of a participant when the research was not previously approved under Subpart C and the investigator believes it is in the best interest of the subject to remain in the study.
- Events that require prompt reporting to the sponsor.
- Complaint of a participant when the complaint indicates unexpected risks or the complaint cannot be resolved by the research team.
- Protocol violation (meaning an accidental or unintentional change to the IRB approved protocol) that placed one or more participants at increased risk, or has the potential to occur again.
- Sponsor-imposed suspension

Unexpected/unanticipated

An event is classified as "unanticipated" when the specificity or severity is not reflected in the study documents including the protocol, or informed consent document.

Related to Study Procedures

The determination of how likely the event is related to the study procedures is made by the principal investigator, the classification of which may vary. The IRB asks if the event is "more likely than not" related to the study procedures.

Involved Risk to Participants or Others

"Participants or others" may involve anyone, including research subjects, research staff, or others not directly involved in the research. The unanticipated problem can occur in either clinical or non-clinical research. "Risks" include physical, psychological, economic, legal or social consequences.

12.4.1.3.1.2. Defining Serious Adverse Events

According to the *Penn Manual for Clinical Research*, and as defined by the Food and Drug Administration, serious adverse events are defined by one of the following:

- Death
- Life-threatening experience
- Inpatient hospitalization or prolongation of hospitalization
- Persistent or significant disability/incapacity
- Congenital anomaly/birth defect in the subject's offspring
- An important medical event that, based upon appropriate medical judgment, may jeopardize the subject and may require medical or surgical intervention to prevent one of the outcomes listed above.

12.4.1.3.1.3. Periodic Surveillance of Adverse Events

Participants in the intervention will be monitored throughout their participation and will be in regular contact with the study team.

12.4.1.3.1.4. Adverse Event Reporting

Safety-related events will be reported in a timely fashion as required by the Data and Safety Monitoring Board and the local IRB. The study principal investigators will be directly responsible for identifying and reporting all serious adverse events, protocol deviations/violations and unanticipated events to the IRBs and funding agencies promptly, as appropriate. Additionally, the DSMB will receive monthly reports of SAEs.

12.4.1.3.1.5. Expected Events

Over the duration of the study, a number of medical events may be expected to occur in adults with CHF, including readmission for CHF exacerbation, surgeries and procedures, the development of cancer or chronic conditions, new or increased symptoms from a chronic condition, musculoskeletal problems.

12.4.1.4. Data Safety Monitoring Board (DSMB)

A Data and Safety Monitoring Board is an independent group of experts convened to protect the safety of research subjects and to ensure that the scientific goals of the project are being met.

This study has been determined to present minimal risk to participants. However, we have established a Data Safety and Monitoring Board of experts in statistics and congestive heart failure to monitor adverse events that occur with study participants. Every 3 to 6 months, all readmissions information is pulled from the UPHS EMR and two residents independently adjudicate every hospitalization. If there is discordance between adjudications, our heart failure expert, Dr. Lee Goldberg, makes the final adjudication. These reports are presented to the DSMB (blinded by arm) so that they can then be assessed for safety. The DSMB report (blinded and de-identified) and DSMB assessments will be

submitted to the IRB at the time of Continuing Review.

12.4.1.4.1. DSMB Responsibilities

The DSMB will perform several duties:

- They will review and approve the research protocol and plans for data and safety monitoring prior to the study.
- They will evaluate the progress of the trial. This will include assessment of data quality, participant recruitment, accrual and retention, participant risk versus benefit, performance of trial sites, and study outcomes. This assessment will be performed at meetings every 6 months during the clinical trial and more frequently if needed
- DSMB reports of adjudicated readmissions will be assessed by the Chair of the DSMB every quarter and reviewed in summary by the full DSMB every six months.
- They will make recommendations to ensure that all of the issues above are appropriately addressed. Study PIs will be responsible for responding to all recommendations of the DSMB and submitting DSMB reports to the IRB.

12.4.1.4.2. DSMB Membership and Affiliation

The DSMB will be composed of experts in cardiology, clinical trials, epidemiology, general internal medicine, and biostatistics, along with project PIs, Drs Kevin Volpp and David Asch from the University of Pennsylvania and statistician Dr. Andrea Troxel from New York University as non-voting members. The PIs will be responsible for maintaining communication between the DSMB and the individual project staff. We consider the proposed trial to be relatively low risk.

Therefore, we have arranged for a monitoring committee that is assigned to review the study and staff training protocols, monitor the trial for safety and adverse events, and conduct quarterly reviews and convene bi-annual meetings. These members will not be involved directly with the trial and have no financial, scientific, or other conflict of interest with the trial. The following individuals have accepted positions as part of the DSMB:

- Patrick Heagerty, PhD – Dr. Heagerty is Professor, Biostatistics and Chair of Biostatistics at the University of Washington. Dr. Heagerty has extensive experience in the conduct, analysis and interpretation of clinical trials. He provides leadership to the CTSA-funded Center for Biomedical Statistics (CBS) through which biostatistical collaboration in the health sciences is coordinated. In his role as director of CBS, Dr. Heagerty leads data coordinating centers for the evaluation of surgical interventions, medical procedures, and for clinical delivery modalities.
- Lynne Warner Stevenson, MD (DSMB Chair) – Dr. Stevenson is Senior Physician Cardiologist at the Brigham and Women’s Hospital and Professor of Medicine at Harvard Medical School. Dr. Stevenson has been the PI or Co-PI of multiple trials examining interventions to improve the management of congestive heart failure. She was the PI on the NHLBI ESCAPE trial as well as one of eight PIs for the first NHLBI Heart Failure Network.
- William Yancy, MD - Dr. William Yancy is Associate Professor of Medicine at Duke University Medical School. Dr. Yancy is an experienced clinical trialist. Dr. Yancy has been PI or Co-PI of multiple clinical trials of diet interventions and co-investigator for numerous other lifestyle intervention trials with such diverse and clinically meaningful outcomes as weight, blood pressure, serum lipids, glycemia, and health-related quality of life.

12.4.1.4.3. Board Process

At the first meeting the DSMB will discuss the protocol, suggested modifications, and establish guidelines for study monitoring by the Board. The Project Manager, in consultation with the Principal Investigators as needed, will prepare the agenda via PPT presentation to provide a study overview, review recruitment methods, and proposed methods for the identification and reporting of adverse events.

Meetings of the DSMB will be held as conference calls as determined by the Chairperson. An emergency meeting of the DSMB may be called at any time by the Chair, should participant safety questions or other unanticipated problems arise. The open portion of the DSMB meetings are attended by the Principal Investigators, key study personnel, and the DSMB members. The closed session is attended by the project leader and the DSMB members.

12.4.1.4.4. Meeting Format

Since the study is open-label, DSMB meetings will consist of open sessions. Discussion held in all sessions is confidential. The Principal Investigator and key members of the study team will attend. Discussion will focus on the conduct and progress of the study, including participant accrual, protocol compliance, and problems encountered.

If necessary, an *executive session* will be attended by voting DSMB members. The executive session will be held to identify and discuss the DSMB's recommendations. The study staff may be present, at the request of the DSMB, during the executive session.

Each meeting must include a recommendation to continue or to terminate the study made by a formal DSMB majority or unanimous vote. Should the DSMB decide to issue a termination recommendation, the full vote of the DSMB is required. In the event of a split vote, majority vote will rule and a minority report should be appended. The DSMB Chair provides the tiebreaking vote in the event of a 50-50 split vote.

A recommendation to terminate the study may be made by the DSMB at any time by majority vote. The Chair should provide such a recommendation immediately by telephone and email to the NIH Program Official. After the NIH Program Official makes a decision about whether to accept or decline the DSMB recommendation to terminate the study, the PI is immediately informed about his decision.

12.4.1.4.5. Meeting Materials

DSMB report templates will be prepared by the study staff, to be reviewed by the DSMB members at each meeting. Format and content of the reports should be reviewed and approved after the first event report is provided, after which modifications to the template may be requested by the Board throughout the trial.

The reports will list and summarize safety data and describe the status of the study. All meeting materials will be sent to the DSMB members prior to each meeting.

Reports will describe participants screened, enrolled, completed, and discontinued, as well as baseline characteristics of the study population. Other general information on study status may also be presented. Listings of deaths, hospitalizations and cause, adverse events and serious adverse events as

well as any other information requested by the DSMB will also be in the report; these data will be presented in a blinded manner. In addition, an unblinded report of adverse events will be prepared by one staff member and available to the DSMB during a closed session, at which the Principal Investigators and key study staff will not be present. The DSMB may request additions and other modifications to the reports on a one-time or continuing basis. The DSMB chair will summarize the closed portion of the meeting in an addendum to be kept confidential until the close of the study.

12.4.1.4.6. Reports from the DSMB

A formal report containing the recommendations for continuation or modifications of the study will be prepared by the DSMB Chairperson. The draft report will be sent to the DSMB members not later than four weeks after the meeting. Once approved by the DSMB members, the DSMB Chair will forward the formal DSMB recommendation to the Principal Investigator. It is the responsibility of the Principal Investigator to distribute the DSMB recommendation to all co-investigators and to ensure that copies are submitted to all the IRBs associated with the study.

As previously stated, the formal DSMB report must include a recommendation to continue or to terminate the study. This recommendation should be made by formal majority vote. A termination recommendation may be made by the DSMB at any time by majority vote. The NIH is responsible for notifying the Principal Investigator of a decision to terminate the study. In the event of a split vote in favor of continuation, a minority report should be contained within the regular DSMB report. The report should not include unblinded data or discussion of the unblinded data.

12.4.1.4.7. Confidentiality

All materials, discussions and proceedings of the DSMB are completely confidential. Members and other participants in DSMB meetings are expected to maintain confidentiality.

PROTOCOL CHANGES LOG

Summary of Amendments and Modifications:

There were three amendments to the Data Analysis plan, motivated by an evolving understanding of disease management pathways for heart failure patients that impacted how to accurately measure the intervention's effectiveness. The first amendment to the Data Analysis plan in November of 2016 was motivated by the addition of censoring criteria for enrolled patients who received an LVAD or transplant during study period because the disease trajectory and treatment paradigm change significantly with these events. It was determined these patients should be censored at the time of LVAD or transplant. The decision to include censoring criteria led to increasing the sample size from 500 to 566 when it was determined the censoring rate was higher than expected. The second amendment to the Data Analysis plan in April 2017 clarifies that the Anderson-Gill formulation of the Cox proportional hazards model will be used to account not just for time to first admission, but also repeated events within individuals to accurately measure the impact of all admissions at the individual level over the course of the intervention. The third amendment was in April 2020 and added a secondary endpoint of cause-specific rehospitalization, the rationale being that evaluating cause-specific hospital admission will allow a more complete understanding of the potential effects of the intervention, by indicating how many of the readmissions are due to what cause (e.g., heart failure, MI, stroke, other cardiovascular cause, non-cardiovascular cause, etc). Other protocol modifications involved minor changes to survey instruments and consent procedures; adding and deleting key personnel to the study team, and the decision to include a qualitative component to the evaluation.

File Name	Date of Submission	Approval date	Summary	Rationale
Mod4	4/6/16	4/24/16	Original IRB protocol submission	Initial trial protocol submission
Mod7	5/12/16	5/18/16	1- Exclusion criteria added<br>2- Noora Marcus, Ben Rosenbach, Michael Kopinsky, Aaron Leitner added as Key Study Personnel<br>3- Updates to study procedures to include device set up instructions	1- In consult with clinical co-investigators, it was determined that patients who were being evaluated for a ventricular assist device or transplant should be excluded as evaluation of these in the medical records indicates likely chance patients will move forward with these treatments and therefore on a different clinical pathway than heart failure management.<br>2- Noora Marcus, Project Manager; Ben Rosenbach, Michael Kopinsky Aaron Leitner, Way to Health programmers<br>3- Edits were made to patient-facing materials for pill bottle and scale device set-up
**File Name**	**Date of Submission**	**Approval date**	**Summary**	**Rationale**

Mod8	5/25/16	5/31/16	1- Exclusion criteria added<br>2- Judith Alvarez removed and Shivani Bhatt and Alyssa Yeager added as Key Study Personnel<br>3- Added motivational messaging to study procedures<br>4- Addition of hospital flyer	1- Exclusion criteria added to exclude potential participants who have a history of heart transplant as clinical pathway is different than heart failure management<br>2- Shivan Bhatt, Clinical Research Coordinator; Alyssa Yeager, Penn medical student who screened potentially eligible patients for further eligibility<br>3- Inspirational/motivational messages will be sent to intervention participants at random timepoints throughout the 12 months to encourage healthy behaviors.<br>4- Recruitment flyer developed to be posted in hospital floors with heart failure patients
Mod9	06/14/16	06/17/16	Control letter added	Letter sent to Control group if patient was randomized to control arm
Mod10	06/15/16	06/19/16	Deviation: Enrollment of ineligible patient due to already on telehealth	Patient should have been excluded from enrollment due to ineligibility criteria
Mod11	08/21/16	8/23/16	Lauren Iannotte added as Key Study Personnel	Lauren Iannotte, Clinical Research Coordinator
Mod12	9/27/16	10/6/17	Deviation: multiple weight or non-adherence alerts not sent from W2H to PennChart	A technical issue in Way to Health (W2H) prevented 7 weight alerts to not transmit into the patients' medical record. Once discovered this was corrected and regular QA review was put in place to prevent future occurrence.
Mod13	9/30/16	10/13/16	Added extended non-adherence letters to patients & providers	Letters sent to participants and providers if participants were non-adherent and non-responsive to multiple phone call attempts

File Name	Date of Submission	Approval date	Summary	Rationale
Mod14	11/7/16	11/22/16	1- Exclusion added for current cognitive or psych condition<br>2- Remove Tirza Calderon and added John	1- Comprehensive exclusion criteria added for "current cognitive or psychiatric condition" which will include: psychosis - schizophrenia/schizophreniform disorder,

			Bergandino to Key Study Personnel<br>3- Added censoring criteria for LVAD & transplant to analysis plan	schizoaffective disorder; severe cognitive impairment; suicidal ideation/attempt; mania; substance abuse; dementia<br>2- John Bergandino, Penn medical student who screened potentially eligible patients for further eligibility<br>3- Censoring criteria added to analysis plan for enrolled patients who receive an LVAD or transplant during study period because the disease trajectory and treatment paradigm change significantly with these events. These patients will be censored at the time of LVAD or transplant; the admission to conduct the transplant will count as an event, but follow-up will cease then and patients will be removed from the risk set.
Mod16	2/2/17	2/15/17	1- Update consent and protocol, data disclosures for Adheretech<br>2- Added new device materials<br>3- Changed Andrea Troxel from Key Study Personnel to advisory	1- Unreliable study devices prompted switch to a more reliable pill bottle device and scales system. This required protocol and consent changes to include data disclosure for Adheretech.<br>2- New patient-facing device materials were created, including changes to welcome letter.<br>3- Andrea Troxel left UPenn for NYU.
Mod17-CR	4/10/17	4/18/17	Continuing Review	Annual Penn IRB Continuing Review

Remote monitoring and behavioral economics in managing patients discharged from the hospital with heart failure: a randomized clinical trial

File Name	Date of Submission	Approval date	Summary	Rationale
Mod18	5/11/17	5/19/17	1- Revised Analysis Plan<br>2- Add Sophia Anderson to Key Study Personnel	1- Analysis plan was updated to 1) increase the overall sample size to 566 to account for censored patients 2) clarify that the Anderson-Gill formulation of the Cox proportional hazards model will be used to account not just for time to first admission, but also repeated events within individuals and 3) updates to censoring plan since some subjects may receive a heart transplant during the course of the study. Because the disease trajectory and treatment paradigm change significantly with that event, patients will be censored at the time of transplant; the admission to conduct the transplant will count as an event, but follow-up will cease then and patients will be removed from the risk set.<br>2- Sophia Anderson, Clinical Research Coordinator
Mod19	5/22/17	5/24/17	Annabel Frank added to Key Study Personnel	Annabel Frank, Penn medical student who screened potentially eligible patients for further eligibility
Mod20	06/28/17	6/28/17	Louisa Whitesides & Melanie Muzelik added as Key Study Personnel	Louisa Whitesides and Melanie Muzelik, Penn Medicine residents who adjudicated readmissions for DSMB report
Mod21	07/18/17	7/25/17	Michael Josephs added to Key Study Personnel	Michael Josephs, Clinical Research Coordinator

File Name	Date of Submission	Approval date	Summary	Rationale
Mod 22 - Deviation	12/1/17	12/11/17	Deviation – missing EPIC alerts	After QA review, it was discovered that there were three issues where weight gains alerts that should have been transmitted to EPIC (PennChart) from Way to Health (W2H) were not transmitted due to user error. Per study protocol, if a study participant steps on a home monitoring scale and triggers either a 3lb in 24 hour weight gain alert or 5lb in 72 hour weigh gain alert, study staff calls the participant to verify weight gain is accurate and then sends an alert from the WTH platform to the patient's EPIC record that is routed to care team. These three failed to transmit due to data entry error for proper routing. All alerts will be checked weekly going forward to make sure they are transmitted properly.
Mod23	01/15/18	01/23/18	Louise Russell added, Shivani Bhatt removed as Key Study Personnel	Louise Russel, co-I for Cost Effectiveness
Mod25	3/19/18	4/2/18	1- Modification of Data Safety and Monitoring Plan to include adjudication plan for all participant readmissions at Penn<br>2- Michael Randazzo added and Devon Taylor removed from Key Study Personnel	1. This study has been determined to present minimal risk to participants. However, we have established a Data Safety and Monitoring Board of experts in statistics and congestive heart failure to monitor adverse events that occur with study participants. Every 3 to 6 months, all readmissions information is pulled from the UPHS EMR and two residents will independently adjudicate every hospitalization. If there is discordance between adjudications, our heart failure expert, Dr. Lee Goldberg, makes the final adjudication. These reports are presented to the DSMB (blinded by arm) so that they can then be assessed for safety. The DSMB report (blinded and de-identified) and DSMB assessments will be submitted to the IRB at the time of Continuing Review.<br>2. Michael Randazzo, Penn medical student who screened potentially eligible patients for further eligibility
File Name	Date of Submission	Approval date	Summary	Rationale

Mod26 - CR	4/10/18	4/16/18	Continuing Review	Annual Continuing Review
Mod27	11/14/18	12/21/18	1- Remove Noora Marcus, John Bergandino and W2H personnel as Key Study Personnel<br>2- Minor clarifications to study procedures to reflect earlier modifications	1- Previous approval to increase sample size to 566 was corrected in two sections so consistent throughout. References to in- person recruitment were removed from study procedures as all recruitment is now done post-discharge over the phone to avoid patient confusion and not interfere with clinical care while patient was still hospitalized.
Mod28	2/1/19	2/7/19	1- Tamar Klaiman added to Key Study Personnel,<br>2- Patient qualitative interview script and protocol added	1- Tamar Klaiman, Sr. Qualitative Research Scientist<br>2- A qualitative component is being added to the study to: 1) identify motivations for patient adherence or non-adherence in the CHF Empower program, and 2) evaluate differences in motivations toward adherence between participants who experienced readmissions during their enrollment period and those who did not.
Mod29	2/22/19	3/13/19	1- Patient qualitative interview script and protocol refined<br>2- Clarification to describe how state-based primary outcomes data will be obtained and linked with Penn Medicine data	1- Several open ended questions were added to the beginning of the interview protocol to elicit further responses about the participant's health status before, during and after their participation in the study. Changes were made throughout to elicit more open ended feedback<br>2- To ensure collection of state-based discharge data from Pennsylvania, New Jersey and Delaware readmissions that occur outside of Penn Medicine are captured for outcomes
Mod30	3/15/19	3/19/19	Addition of invitation letter for patient qualitative interviews	Implementation of a letter to send to eligible, enrolled intervention participants to remind them they can participate in a 45-minute phone interview at the end of their participation in the study.
**File Name**	**Date of Submission**	**Approval date**	**Summary**	**Rationale**
Mod31 -	3/31/19	4/5/19	Continuing Review	Annual continuing review

CR				
Mod32	6/11/19	6/20/19	1- Remove Savannah Aepli, add William Ferrell and Bryson Houston as Key Study Personnel<br>2- Edits to patient qualitative interview guide	1- William Ferrell, Clinical Research Coordinator PT help for patient monitoring; Bryston Houston, SUMR scholar working on alert analysis<br>2- The semi-structured interview guide questions/probes script has been edited to more accurately reflect the flow of conversation.
Mod33	8/9/19	8/21/19	1- Erin Huang added and Sophia Anderson removed as Key Study Personnel<br>2- Provider qualitative interview guides and protocol added	1- Erin Huang, Data Analyst for pulling PennChart patient outcome data; initiation of provider qualitative interviews<br>2- A qualitative component for providers is being added to the study to assess the impact of the CHF Empower program on providers' perceptions of how easy the program was to use, the program's impact on patient care, and the effect on patients' health.
Mod34 – Deviation	1/13/20	2/12/20	Akriti Mishra added and removed as Key Study Personnel	Corrected omission of Akrita Mishra, Data Analyst for pulling PennChart outcome data. Akriti had not been added (or subsequently removed) as Key Study Personnel in the IRB Protocol. Akriti Mishra worked on the study starting in March of 2018 and stopped working on the study in August of 2019 when she left UPenn for another position. This omission of adding and removing Akriti Mishra as Key Study Personnel was an oversight by the Project Director. Akriti Mishra was tasked specifically with occasional (biannual) data pulls of hospital readmissions for the enrolled study participants. The data pulls necessarily involved the viewing and handling of data with PHI. Akriti then securely transferred these data pulls to the study team in order for them to prepare summary reports for the Data and Safety Monitoring Board to assess study safety.
**File Name**	**Date of Submission**	**Approval date**	**Summary**	**Rationale**
Mod35	2/13/20	2/19/20	Add Jingsan Zhu added and Bryson Houston removed as Key Study Personnel	Jingsan Zhu, Data Director overseeing all study analyses
Mod36 –	3/10/20	3/31/20	Continuing Review	Annual Continuing Review

CR				
Mod37	4/1/20	4/2/20	Yuqing Lin added as Key Study Personnel	Yuqing Lin, Data Analyst for study outcomes
Mod38	4/22/20	4/24/20	Amended secondary outcome variable to include “assess cause-specific rehospitalizations”	A secondary endpoint will be cause-specific rehospitalization, the rationale being that evaluating cause-specific hospital admission will allow a more complete understanding of the potential effects of the intervention, by indicating how many of the readmissions are due to what cause (e.g., heart failure, MI, stroke, other cardiovascular cause, non-cardiovascular cause, etc).
Mod39	5/14/20	5/18/20	Chris Snider added as Key Study Personnel	Chris Snider, Data Analyst for CareEverywhere outcome data
Mod40	6/17/20	6/25/20	1- Jiali (Helen) Yan added as Key Study Personnel.<br>2- Change to Subject Confidentiality section in protocol to clarify how the NJDOH inpatient data will be matched to study participants<br>:<br>.	1- Jiali Yang, Data Analyst for CareEverywhere outcome data<br>2- In Subject Confidentiality, the following has been added to clarify the process: For obtaining NJDOH state-based hospitalization data, NJDOH will send state-wide inpatient data from 2016-2020 with name, DOB, address, gender, race, and ethnicity. Our data analysts will match on these variables to the enrolled participants.
**File Name**	**Date of Submission**	**Approval date**	**Summary**	**Rationale**
Mod41	6/29/20	6/30/20	Subject Confidentiality section of protocol updated to include review of CareEverywhere data	Participants in this study consent to having their medical care reviewed during the period they are enrolled in the study (365 days). This includes reviewing inpatient medical information contained in PennChart (all participants were Penn Med patients at the time of enrollment) as well as state-based inpatient data from NJ, DE and PA to capture re-hospitalizations that may have occurred outside of Penn Medicine. This modification is to clarify that we will also plan to review the CareEverywhere data that

				further details the clinical reasons for readmissions that occur outside Penn Medicine and is already a part of the enrolled participants' PennChart medical record. The information contained in CareEverywhere (combined with the state based inpatient data) is crucial to assessing the main outcome of the study by ensuring we are accurately assessing the clinical reasons enrolled participants are being readmitted and that we are doing this assessment using the same procedures used to assess the readmissions that occur at Penn Medicine.

Original Statistical Analysis Plan

Statistical considerations

Power and sample size issues. Our primary outcome is time to first readmission for any cause. The primary comparison will be the time to event compared between the intervention arm and the usual care arm; that is, the primary hypothesis is whether a series of interventions, adapted to the behavior and outcomes of each particular patient, will reduce readmission rates compared to usual care.

We expect the 1-year event rate to be 47% in the usual care arm based on data obtained on our target population at UPHS. We wish to have 80% power to detect an absolute decrease in this rate of about 10% in the intervention group; this corresponds to a hazard ratio of 0.73. We will accrue 500 patients over the 24-month enrollment period and follow them for an additional 12 months; with the 1:1 randomization ratio, we will have 250 control subjects and 250 intervention subjects. The primary hypothesis will use a two-sided Type I error rate of 0.05.

Data analysis plans

Prior to analysis, we will produce data summaries including graphical methods to assess data quality, examine central tendencies and distributional assumptions and randomization success. The primary analysis will consist of unadjusted intent-to-treat hypothesis tests using the logrank test to compare the time to hospitalization in the two groups. We will also estimate Cox proportional hazards regression models adjusted for the stratification variable and other covariates of interest (such as patient sex, income, race, baseline ejection fraction, and quality of life), retaining these given evidence of confounding or predictive ability. We will employ a confounder selection method based on "change in estimate" criterion. We will assess interaction terms between the a priori potential effect modifiers such as income level, race, and baseline ejection fraction. All hypothesis tests will be two-sided and models will be assessed using standard diagnostic techniques. We will use standard approaches to assess the proportional hazards assumption and include interaction terms with time if necessary. We will also fit frailty models to analyze repeated rehospitalizations within patients; these models properly adjust for the correlation of multiple events within individuals.

Handling of missing data is an important issue in all RCTs. Follow-up data may be missing if participants miss the follow-up visit or withdraw. . Even in subjects who are lost to follow-up, however, we will be able to capture any events that result in hospitalization, and thus will still be able to observe our primary outcome. We will compare dropout rates by arm, will attempt to find the reasons for missing data and will compare baseline characteristics in participants with complete vs. incomplete follow-up. In secondary analyses we will investigate the sensitivity to modeling assumptions using imputation models and inverse-probability-weighted estimating equations and models that adjust for informative missing data.

Cost effectiveness analysis

To assess the cost-effectiveness of the interventions, we will use analytic methods for economic evaluations in clinical trials. Our approach will be similar to Specific Aim 3 using cost as the outcome. We will use generalized linear models to adjust for the stratification variable and other factors. Cost-effectiveness ratios will be calculated as the difference in costs divided by the difference in rehospitalization rate calculated under Specific Aim 3 for the “within-trial” analysis, with parametric 95% CIs for the cost per percentage point increase in adherence and acceptability curves. Standard errors and the correlation of the difference in cost and effect will be obtained using a bootstrap procedure. A

further cost-effectiveness analysis from the “societal perspective” will be conducted to assess the impact of the rehospitalization rate reductions measured as cost per QALY gained. To address uncertainty in the micro simulation model, we will also conduct a probabilistic sensitivity analysis (PSA162) by defining probability distributions for the variables in the model used to calculate costs and effectiveness.163 We will use the results of the PSA to calculate confidence (or credible) intervals and acceptability curves.

Final Statistical Analysis Plan

Statistical considerations

Power and sample size issues. Our primary outcome is time to readmission for any cause. The primary comparison will be the times to events compared between the intervention arm and the usual care arm; that is, the primary hypothesis is whether a series of interventions, adapted to the behavior and outcomes of each particular patient, will reduce readmission rates compared to usual care.

We expect the 1-year event rate to be 47% in the usual care arm based on data obtained on our target population at UPHS. We wish to have 80% power to detect an absolute decrease in this rate of about 10% in the intervention group; this corresponds to a hazard ratio of 0.73. We will accrue 566 patients over the 24-month enrollment period and follow them for an additional 12 months; with the 1:1 randomization ratio, we will have approximately 283 subjects in each arm. The primary hypothesis test will use a two-sided Type I error rate of 0.05. Note that the power calculations are based on the time to first readmission; if patients experience multiple readmissions, statistical power will be increased.

Data analysis plans

Prior to analysis, we will produce data summaries including graphical methods to assess data quality, examine central tendencies and distributional assumptions and randomization success. The primary analysis will consist of an unadjusted intent-to-treat hypothesis test using the Andersen-Gill formulation of the Cox proportional hazards model to compare the times to hospitalizations in the two groups; these models properly adjust for the correlation of multiple repeated events within individuals.. We will also estimate multivariate regression models adjusted for the stratification variable and other covariates of interest (such as patient sex, income, race, baseline ejection fraction, and quality of life), retaining these given evidence of confounding or predictive ability. We will employ a confounder selection method based on "change in estimate" criterion. We will assess interaction terms between the a priori potential effect modifiers such as income level, race, and baseline ejection fraction. All hypothesis tests will be two-sided and models will be assessed using standard diagnostic techniques. We will use standard approaches to assess the modeling assumptions and include interaction terms with time if necessary.

Handling of missing data is an important issue in all RCTs. Follow-up data may be missing if participants miss the follow-up visit or withdraw. Even in subjects who are lost to follow-up, however, we will be able to capture any events that result in hospitalization, and thus will still be able to observe our primary outcome. In addition, some subjects may receive a heart transplant during the course of the study. Because the disease trajectory and treatment paradigm change significantly with that event, we will censor patients at the time of transplant; the admission to conduct the transplant will count as an event, but follow-up will cease then and patients will be removed from the risk set. We will compare dropout rates by arm, will attempt to find the reasons for missing data and will compare baseline characteristics in participants with complete vs. incomplete follow-up. In secondary analyses we will investigate the sensitivity to modeling assumptions using imputation models and inverse-probability-weighted estimating equations and models that adjust for informative missing data.

A secondary endpoint will be cause-specific rehospitalization, the rationale being that evaluating cause-specific hospital admission will allow a more complete understanding of the potential effects of the intervention, by indicating how many of the readmissions are due to what cause (e.g., heart failure, MI, stroke, other cardiovascular cause, non-cardiovascular cause, etc).

Cost effectiveness analysis

To assess the cost-effectiveness of the interventions, we will use analytic methods for economic evaluations in clinical trials. Our approach will be similar to Specific Aim 3 using cost as the outcome. We will use generalized linear models to adjust for the stratification variable and other factors. Cost-effectiveness ratios will be calculated as the difference in costs divided by the difference in rehospitalization rate calculated under Specific Aim 3 for the "within-trial" analysis, with parametric 95% CIs for the cost per percentage point increase in adherence and acceptability curves.97 Standard errors and the correlation of the difference in cost and effect will be obtained using a bootstrap procedure. A further cost-effectiveness analysis from the "societal perspective" will be conducted to assess the impact of the rehospitalization rate reductions measured as cost per QALY gained. To address uncertainty in the micro simulation model, we will also conduct a probabilistic sensitivity analysis (PSA162) by defining probability distributions for the variables in the model used to calculate costs and effectiveness. We will use the results of the PSA to calculate confidence (or credible) intervals and acceptability curves.

Summary of statistical analysis plan changes

There were three amendments to the Data Analysis plan, motivated by an evolving understanding of disease management pathways for heart failure patients that impacted how to accurately measure the intervention's effectiveness. The first amendment to the Data Analysis plan in November of 2016 was motivated by the addition of censoring criteria for enrolled patients who received an LVAD or transplant during study period because the disease trajectory and treatment paradigm change significantly with these events. It was determined these patients should be censored at the time of LVAD or transplant. The decision to include censoring criteria led to increasing the sample size from 500 to 566 when it was determined the censoring rate was higher than expected. The second amendment to the Analysis plan in April 2017 clarifies that the Anderson-Gill formulation of the Cox proportional hazards model will be used to account not just for time to first admission, but also repeated events within individuals to accurately measure the impact of all admissions at the individual level over the course of the intervention. The third amendment was in April 2020 and added a secondary endpoint of cause-specific rehospitalization, the rationale being that evaluating cause-specific hospital admission will allow a more complete understanding of the potential effects of the intervention, by indicating how many of the readmissions are due to what cause (e.g., heart failure, MI, stroke, other cardiovascular cause, non-cardiovascular cause, etc).
