# Supplementary material for: Remote Monitoring and Behavioral Economics in Managing Heart Failure in Patients Discharged From the Hospital: A Randomized Clinical Trial
Source: JAMA Intern Med. 2022 May 9;182(6):643–9. doi: 10.1001/jamainternmed.2022.1383 (PMC9171555; doi:10.1001/jamainternmed.2022.1383)
Supplement: Supplement 2. — eFigure 1. Patient Weight Change Since Index Hospitalization by Study Group eFigure 2. Mean Weekly Adherence to Medication and Weigh-ins for Intervention Group Patients eTable 1. Detailed List of Participant Exclusions eTable 2. Event Rates According to Degree of Adherence eTable 3. Association Between Alerts and Cardiovascular Events at 1, 2, and 4 Weeks for Intervention Group Patients eTable 4. Noncardiac Reasons for Admission Among Intervention and Control Group Patients [file jamainternmed-e221383-s002.pdf]

Supplementary Online Content

Asch DA, Troxel AB, Goldberg LR, et al. Remote monitoring and behavioral economics in managing heart failure in patients discharged from the hospital: a randomized clinical trial. *JAMA Intern Med*. Published online May 9, 2022. doi:10.1001/jamainternmed.2022.1383

eFigure 1. Patient Weight Change Since Index Hospitalization by Study Group

eFigure 2. Mean Weekly Adherence to Medication and Weigh-ins for Intervention Group Patients

eTable 1. Detailed List of Participant Exclusions

eTable 2. Event Rates According to Degree of Adherence

eTable 3. Association Between Alerts and Cardiovascular Events at 1, 2, and 4 Weeks for Intervention Group Patients

eTable 4. Noncardiac Reasons for Admission Among Intervention and Control Group Patients

This supplemental material has been provided by the authors to give readers additional information about their work.

eFigure 1. Patient weight change since index hospitalization by study group

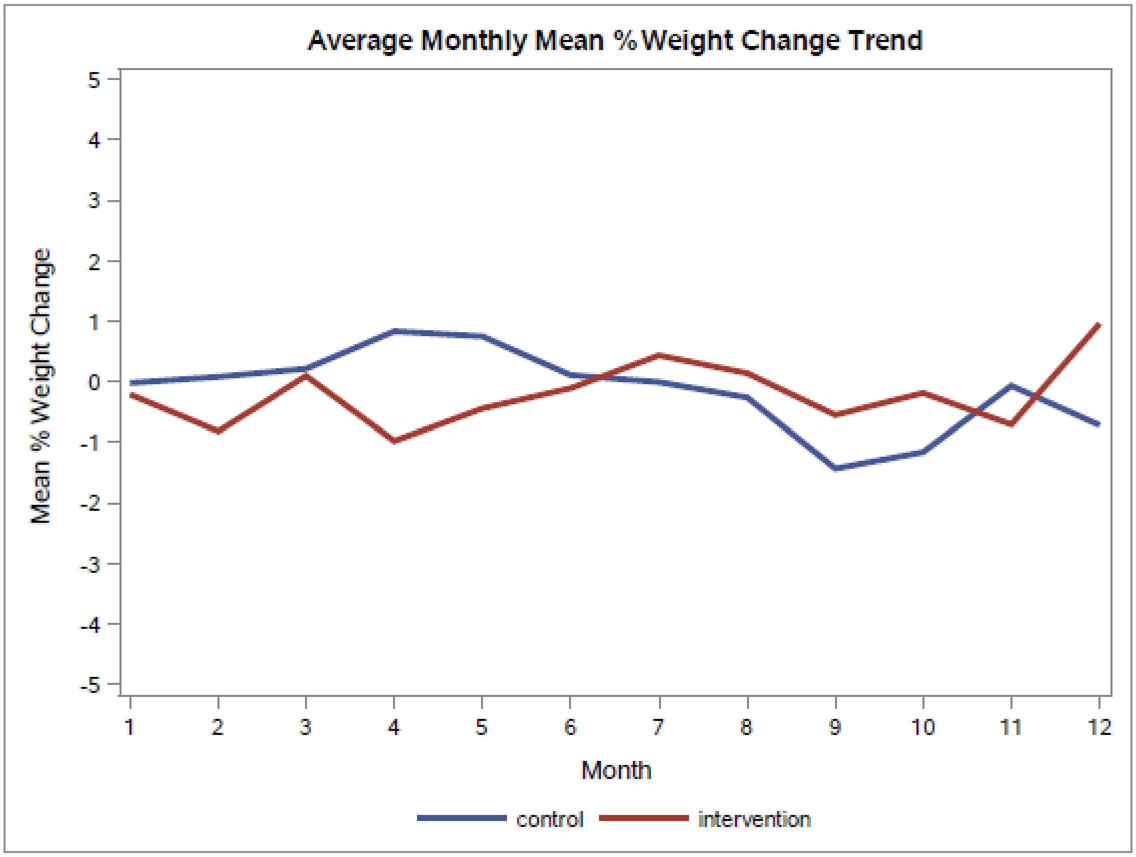

Month	1	2	3	4	5	6	7	8	9	10	11	12
No. patients												
Control	280	207	179	165	156	162	144	142	132	143	131	131
Intervention	272	196	159	152	162	139	137	132	121	113	113	114

Note: Limited to patients with weight data in UPHS electronic record. Weight data was collected from outpatient encounters or within the first two days of inpatient encounters. Not all patients generated weight data every month. Weight change is compared to weight measured on or near index discharge date.

eFigure 2. Mean weekly adherence to medication and weigh-ins for intervention group patients

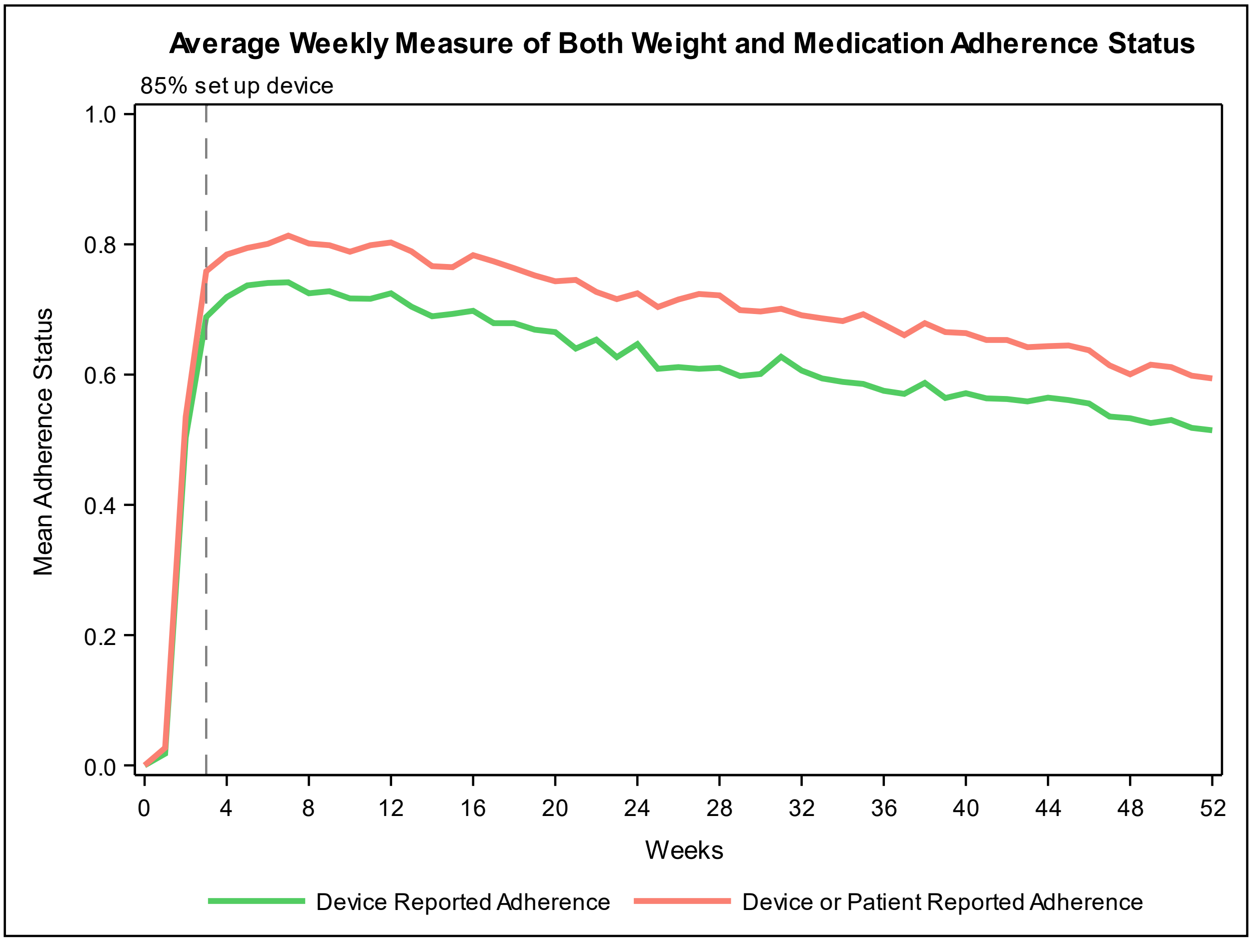

Dotted line indicates the number of weeks by which 85% of patients set up the scale and pill bottle. *Device Reported Adherence* includes only data directly submitted by devices (wireless pill bottle and scale). *Device or Patient Reported Adherence* includes data directly submitted by devices as well as patient self-report, and therefore is higher. Days patients were in hospital were excluded from the calculation of adherence rate

eTable 1. Detailed list of participant exclusions

Category	Variables included
Death	Death
Not 18-80	Age
No CHF or diuretic	No CHF<br>CHF not primary problem or final discharge diagnosis<br>No diuretic<br>Non-daily diuretic
Administrative conflicts	No UPHS follow up<br>Not inpatient visit (aka not admitted)<br>Not discharged home<br>Inability to consent due to language or disability
Medical exclusion	Medically unstable<br>Mentally unstable<br>Dementia<br>Metastatic cancer<br>Palliative care<br>Mobility issues<br>End-stage renal disease<br>GFR < 25 ml/min<br>Dialysis
Receiving advanced HF support	Heart transplant list/hx<br>Heart transplant eval<br>VAD<br>Inotrope-dependent
Enrolled in conflicting program	Enrolled in pilot<br>CardioMEMs monitor<br>Other telemedicine intervention

eTable 2. Event rates according to degree of adherence

eTable 2a. Event rates according to degree of adherence to both weight measurement and diuretic medication for intervention group patients

	High Adherence [0.8,1] N=128	Medium Adherence [0.5,0.8) N=67	Low Adherence [0,0.5) N=62
Readmissions or deaths per patient - Mean (SD)	0.91 (1.17)	1.85 (1.72)	1.84 (2.27)
Hospital days per patient - Mean (SD)	5.55 (8.75)	15.84 (21.42)	17.62 (26.56)
Days to first event - Mean (SD)*	132 (95)	129.34 (97.8)	98.81 (87.95)
CHF readmissions or deaths per patient - Mean (SD)	0.36 (0.74)	0.64 (0.98)	0.64 (1.11)

SD = standard deviation. * Among patients who experienced defined events of readmission or death

eTable 2b. Event rates according to degree of adherence to weight measurement for intervention group patients

	High Adherence [0.8,1] N=145	Medium Adherence [0.5,0.8) N=66	Low Adherence [0,0.5) N=45
Readmissions or deaths per patient - Mean (SD)	0.96 (1.23)	1.88 (1.73)	2(2.43)
Hospital days per patient - Mean (SD)	5.89 (8.94)	16.52 (22.26)	20.04 (29.07)
Days to first event - Mean (SD)*	132.04 (93.83)	123.84 (101.39)	97.81 (82.15)
CHF readmissions or deaths per patient - Mean (SD)	0.38 (0.74)	0.59 (0.98)	0.76 (1.23)

eTable 2c. Event rates according to degree of adherence to diuretic medication for intervention group patients

	High Adherence [0.8,1] N=161	Medium Adherence [0.5,0.8) N=53	Low Adherence [0,0.5) N=42
Readmissions or deaths per patient - Mean (SD)	0.98 (1.2)	2.08 (1.91)	2.05 (2.43
Hospital days per patient - Mean (SD)	6.37 (9.78)	17.7 (23.8)	21.02 (29.45)
Days to first event - Mean (SD)*	136.22 (94.37)	112.72 (102.31)	94.93 (79.35)
CHF readmissions or deaths per patient - Mean (SD)	0.37 (0.72)	0.68 (1.09)	0.76 (1.21)

eTable 3. Association between alerts and cardiovascular events at 1, 2, and 4 weeks for intervention group patients

	Alert Types					
	Chest Pain or dyspnea	Edema, loss of appetite, nausea, difficulty with medications	Other symptoms	No symptoms reported	Unreachable for symptom check	All alerts
Total number of alerts	259	651	198	1996	632	3736
Number of events within 1 week of alert.	18	14	6	10	17	65
# Events/# Alerts	6.95%	2.15%	3.03%	0.50%	2.69%	1.74%
Number of events within 2 weeks of alert.	25	23	9	46	26	129
# Events/# Alerts	9.65%	3.53%	4.55%	2.30%	4.11%	3.45%
Number of events within 4 weeks of alert.	30	46	13	83	48	220
# Events/# Alerts	11.58%	7.07%	6.57%	4.16%	7.59%	5.89%

Events reflect death or readmissions or hospitalizations or observations stays for cardiovascular reasons.

eTable 4. Noncardiac reasons for admission among intervention and control group patients

	Control<br>193 Admissions	Intervention<br>171 Admissions
Chronic obstructive pulmonary disease/respiratory failure	34	25
Pneumonia	6	13
Other infection/sepsis	22	30
Renal failure	18	20
Gastrointestinal disorders	32	11
Other (e.g., diabetes, post surgical complications, sickle cell disease, cancer)	81	72
