# Supplementary material for: Remote Monitoring and Behavioral Economics in Managing Heart Failure in Patients Discharged From the Hospital: A Randomized Clinical Trial
Source: JAMA Intern Med. 2022 May 9;182(6):643–9. doi: 10.1001/jamainternmed.2022.1383 (PMC9171555; doi:10.1001/jamainternmed.2022.1383)
Supplement: Supplement 3. — Data Sharing Statement [file jamainternmed-e221383-s00.pdf]

Data Sharing Statement

Asch. Remote Monitoring and Behavioral Economics in Managing Heart Failure in Patients Discharged From the Hospital. *JAMA Intern Med*. Published May 09, 2022. doi:10.1001/jamainternmed.2022.1383

Data
Data available: No

Additional Information
Explanation for why data not available: Data essential for the analyses in these studies come from state-regulated data use agreements that prohibit sharing.
